# Epigenetics of generalized internet use disorder including data on overuse of social media: a closer look at the SLC6A4, NR3C1 and OXT genes

**DOI:** 10.1186/s12888-026-08743-1

**Published:** 2026-10-14

**Authors:** Christian Montag, Markus Kiefer, Bernhard J. Connemann, Thomas Kammer, Simon Sanwald

**Affiliations:** 1 Centre for Cognitive and Brain Sciences, Institute of Collaborative Innovation, University Macau, Macau, SAR China; 2 Department of Psychology, Faculty of Social Sciences, University of Macau, Macau, SAR China; 3 Department of Computer and Information Science, Faculty of Science and Technology, University of Macau, Macau, SAR China; 4 Department of Psychiatry and Psychotherapy III, Ulm University, Ulm, Germany

**Keywords:** Epigenetics, SLC6A4, OXT, NR3C1, Problematic social media use, Internet addiction tendencies

## Abstract

**Background:**

Only few studies so far have investigated the molecular underpinnings of Internet Use Disorders (IUD), which can shed light on the neurochemical basis of excessive online behaviors. There are even less epigenetic studies in this research field, and longitudinal evidence is even more scarce.

**Methods:**

The present study investigated in *n* = 83 depressed patients and *n* = 83 healthy controls potential links between DNA-methylation of three candidate genes (serotonin transporter gene SLC6A4, glucocorticoid receptor gene NR3C1 and gene coding for oxytocin OXT) known to play a role in affective disorders and self-reported excessive Internet and problematic social media use over a two-week period.

**Results:**

In depressed patients, we found a significant positive association between problematic social media use, IUD tendencies in general, craving and methylation of a CpG site in NR3C1. In healthy controls, we found a significant positive association between social support and methylation of a CpG site in OXT. Further we found that higher social media addiction tendencies were associated with decreasing differences in methylation of OXT over a two-week period in both the group of depressed patients and healthy controls. However, this effect was small and the confidence interval contained zero.

**Conclusions:**

Our findings might provide first preliminary indications that NR3C1 and OXT might be involved in overuse of social media. However, small effect sizes and complex interaction effects require replication in larger samples with longitudinal measurements over a longer time period.

**Supplementary Information:**

The online version contains supplementary material available at https://doi.org/10.1186/s12888-026-08743-1.

## Background

Internet Use Disorders (IUD; formerly known as Internet addiction) have a global prevalence of approximately 7% (assessed across samples of the meta-analysis, at least when one considers unspecified Internet Use Disorders [48]. The umbrella term IUD comprises different excessive online activities, including gaming, gambling, or overuse of social media [40]. Prevalence estimates for other distinct activities such as “social media addiction” led also to substantial numbers, although no unified framework exists to study this non-officially recognized condition [14]. This said, global discussions about harmful effects of excessive social media and Internet use – in particular also in childhood and adolescence – regularly appear in the mass media and are discussed globally [41]. Therefore, it is of importance to better understand the biological basis of IUDs. This will help characterize this potential new form of behavioral addictions (aside from Gaming Disorder and Gambling Disorder, the WHO does not officially recognize other addictive behaviors as bona fide disorders).

Several vulnerability factors are discussed as being useful to understand who is more likely to develop IUD [11]. According to the Interaction of Person-Affect-Cognition-Execution-variables-(I-PACE) model to predict IUDs, person variables are of particular interest to shed light on such vulnerability factors. The person variable in the I-PACE model comprises a history of psychopathology and personality traits (such as high neuroticism/low conscientiousness) and genetics. A twin study suggests that a genetic cluster linked to the personality trait self-directedness (a character dimension in Cloninger’s biosocial theory of personality) might explain the genetics underlying individual differences in IUD traits due to shared variance [22]. This speaks for the idea that genes linked to IUDs might be those having to do with self-regulation abilities. Although heritability estimates differ across studies, we exemplarily point to the work with 5247 monozygotic and dizygotic twins reporting 48% of a genetic influence [71]. This is also in line with twin studies (and genome wide associations studies) investigating heritability estimates from substance use disorders demonstrating an influence of genetics [16]. After twin studies have established that genetics at least explain some variance of individual differences in IUD, the logical next step is to study the molecular genetic underpinnings of IUDs [43, 73]. This field is still understudied as in more than 15 years only a couple of studies have investigated IUDs and gaming disorder using the candidate gene approach [65]. The findings in the literature on molecular genetic associations with IUDs are not well replicated, which may be related to the investigation of small samples underpowered to detect the tiny effects of single genetic variants [39].

Since environmental and genetic factors impact IUD development [34], the present study aimed to investigate the molecular level, where genes meet environmental stressors, the epigenome [55]. The epigenome represents the level of the molecular study of psychological and psychiatric phenotypes in which the environment meets genetics. In other words, it is well-known that environmental factors can impact upon gene regulation by altering the epigenome [62]. A critical mechanism that is the focus of psychological and psychiatric research is the investigation of methylation patterns in the promoter regions of genes [61]. A candidate gene which has previously been implicated in IUDs is the serotonin transporter gene (SLC6A4) [33], but until now with heterogenous findings [13, 80]. Given the existing attempts to link the serotonin-transporter-polymorphisms with IUDs, the present study aims to investigate the layer above the genetic code of the SLC6A4 gene – hence the epigenome – in the realm of IUDs. The SLC6A4 codes for the serotonin transporter, an often-targeted structure in the brain for treating depression when using antidepressants; for a more nuanced view see a recent work [1]. One epigenetic mechanism is DNA methylation, which refers to the binding of a methyl group to nucleobases [9]. In case of SLC6A4 methylation, higher methylation levels have been shown to be associated with lower levels of SLC6A4 mRNA [54, 72]. This is of interest for the present study, because preliminary evidence suggests that treating patients suffering from IUD with SSRIs – hence the psychopharmaceutical targeting the serotonin transporter – improves the IUD condition (for instance a reduction of symptoms in the context of Internet Gaming Disorder between 17.6% and 24.0% was observed according to a recent review; [60]. As IUDs go along with negative affect, it is noteworthy that a recent meta-analysis found an association between depression and SLC6A4 hypermethylation [82]. However, this association was confined to a subgroup analysis, which does not support the hypothesis of a general SLC6A4 hypermethylation in individuals suffering from depression. Aside from the SLC6A4 gene, we also present exploratory findings on epigenetic information related to NR3C1 and OXT gene. The NR3C1 gene codes for the glucocorticoid receptor and has been implicated in alcohol use disorder: The authors observed an increased methylation of exon variant 1_H_ of the NR3C1 gene in the context of chronic alcohol drinking [19]. Clearly, this does not imply that this gene also plays a role in Internet Use Disorders, but it could be the case due the addictive nature of both excessive Internet and alcohol use, and some studies also suggest a link between IUD and problematic alcohol use [31, 45]. The gene coding for oxytocin (OXT) so far has, to our knowledge, not been studied in the context of addictive behavior, but lower OXT methylation has been associated with higher sociability [20] and, more recently, also depression [61, 63]. This said, a recent epigenetic work implicated the gene coding for the oxytocin receptor (OXTR) to play a role in IUD with showing positive correlation between Internet Addiction Test scores and methylation of several CpG sites on the OXTR gene (but only in subject with IAT scores < 50; [5]. Of note, this group also investigated the epigenetics of the dopamine transporter gene and the serotonin transporter gene in the context of Internet addiction – the findings were strongly dependent on the analysis methods (e.g., which groups with which cut-off-scores were analyzed, etc.).

The present study investigates a healthy sample and a depressed sample over a two-week period. Since psychiatric disorders show significant symptom improvement within the first 14 days of treatment [49], we hypothesized that if methylation change is relevant for inducing early treatment success, we should observe changes in methylation of relevant target genes after a two-week treatment period. Regarding methylation change in NR3C1, SLC6A4 and OXT, the few studies that investigate methylation change observed dynamic methylation patterns anywhere from 45 min to weeks, months and even a year [15, 30, 44, 69, 76]. Hence, data on methylation changes over a two-week treatment period complement the current knowledge on the dynamics of methylation of three target genes. As IUD does not represent an official diagnosis (see above), the present study focused not only on the study of individual differences in a subclinical sample but had the chance to study depressed patients including their IUD tendencies. This might represent a promising approach to study IUDs, because depressive tendencies represent a well-known co-morbidity of Internet Use Disorders including overuse of social media [27, 57]. In the I-PACE model it has also been suggested that a history of depression might more easily pave the way to also experience IUD (as a person variable; [11, 12], which makes the investigation of depressed patients also an interesting research endeavor. Please note that unspecific IUD (such as asking a person on generalized overuse of the Internet) overlaps robustly with overuse of social media [38, 46]. Given the current discussions around the actual nature of overusing social media, we not only investigated generalized IUD tendencies in this work but also overuse of social media to understand how this more specific overuse of the Internet would relate to methylation patterns of the candidate genes. Our main aim was to understand if individual differences in methylation patterns would be correlated with IUD tendencies and if we would see same associations in control persons and depressed patients, with the patients likely representing participants being more prone to experience IUDs. In addition, we investigated how IUD tendencies are associated with changes in methylation of the candidate genes over a two-week period. Our rationale was that trait IUD tendencies could be a predictor of methylation change. This assumption was motivated by previous findings that the presence of IUD tendencies predicts a less successful treatment outcome in adults with depression [81], suggesting that IUD tendencies may also be linked to biological processes accompanying treatment response. Thus, we are, to the best of our knowledge, the first to investigate longitudinal associations of IUD tendencies and methylation of SLC6A4, NR3C1 and OXT. In general, our study should be seen as highly exploratory, because of the few studies in the field so far (hence we need to apply strong procedures for multiple testing).

## Methods

### Participants

Data of *N* = 99 inpatients suffering from Major Depressive Disorder (MDD) and *N* = 169 healthy controls (HC) were collected and stored in a database prior to pre-registration. Data was analyzed after pre-registration (pre-registration: https://osf.io/bkufq). Data of the first measurement point of the current study partly overlaps with data reported in Sanwald et al. [63]. Depressed inpatients were recruited at the Department of Psychiatry and Psychotherapy III at Ulm University, Ulm, Germany. Patients were diagnosed with MDD by a psychiatrist at admission to the hospital using the Structured Clinical Interview for DSM-IV (SCID-IV) (American Psychiatric Association, [2]). *N* = 16 inpatients were excluded, since they were diagnosed with a disorder other than MDD or comorbid anxiety disorders during their stay at the inpatient treatment unit. We calculated dose equivalents for antidepressants (weighted mean dose/fluoxetin40 mg) [24] and antipsychotics (weighted mean dose/Chlorpromazine100 mg) [8, 64, 78]. Table 1 shows medication in the group of inpatients.

The sample of HC was recruited by (online) advertisement. If interested, HC contacted our clinic and were invited to the first measurement. They filled out the questionnaires described below. In addition, HC underwent a diagnostic interview (Mini-DIPS, [36]) and SCID-5-PD [3] to exclude participants if any mental disorder was suspected. *N* = 43 healthy controls were excluded because a mental disorder was indicated by the interview or diagnosed in the past. Covariates were also assessed during the interview. Afterwards, venous blood was taken for analyses of DNA-methylation, blood cell composition and mRNA abundance. Blood samples for blood cell composition were processed within the same workday. Blood samples for DNA-methylation and mRNA abundance were gradually cooled to -80 degrees Celsius and stored at this temperature until they were slowly thawed for methylation and mRNA analysis. HCs were matched to patients by means of a nearest neighbor matching (see Statistical analysis) resulting in a sample size of *N* = 83 individuals per group (MDD: *n* = 48 female; HC: *n* = 48 female). Covariates for matching were assessed by means of a semi-standardized inhouse interview. Alcohol consumption was measured in grams/day and nicotine consumption in cigarettes/day. All procedures performed in this study were approved by the ethics committee of Ulm University, Ulm, Germany (approval date: 10th December 2020, number: 25/18). Written voluntary informed consent of the participants was obtained after the nature of the procedures had been fully explained.

**Table 1:** Therapeutic approaches in the group of MDD inpatients

	T0			T1	
**Antidepressants**	***n***		**%**	***n***	**%**
SSRIs	46		55.4	29	51.8
SNRIs	16		19.3	13	23.2
TCAs	18		21.7	10	17.9
TeCAs	13		15.7	8	14.3
Atypical	6		7.2	8	14.3
**Neuroleptics**	***n***		**%**	***n***	**%**
First generation	2		2.4	1	1.8
Second generation	21		25.3	13	23.2

Note. T0: first measurement point; T1: second measurement point; SSRI: selective serotonin reuptake inhibitors; SNRI: serotonin-norepinephrine reuptake inhibitors; TCA: tricyclic antidepressants; TeCA: tetracyclic antidepressants; ECT: electroconvulsive therapy; BLT: bright light therapy; CBT: cognitive behavioral therapy. Note that some patients had more than one medication and differing percentages are due to drop-outs at t1

### Questionnaires

All participants completed the German version of the short Internet Addiction Test (s-IAT), which provides insights into unspecified IUD tendencies [50, 67]. The questionnaire consists of 12 items answered in a five-answer format ranging from 1 = never to 5 = very often. The items were summed up to obtain a total score, with higher scores indicating higher IUD tendencies. Furthermore, two facet dimensions can be built: loss of control/time management and craving/social problems. Internal consistencies are as follows: HC: *α* = 0.92; MDD: *α* = 0.92.

Aside from the s-IAT, the Bergen Social Media Addiction Scale (BSMAS) was used (Andreassen et al., 2012). It consists of six items answered in a five-item format ranging from 1 (very rarely) to 5 (very often). The items were summed to obtain a total score. The German version was reformulated to assess social media addiction in general (and not just focus on Facebook; [10]. Higher scores indicate higher social network use disorder tendencies. Internal consistencies are as follows: HC: *α* = 0.88; MDD: *α* = 0.88.

Finally, the „Fragebogen zur Erfassung von Ressourcen und Selbstmanagementfähigkeiten“ (FERUS)/Social Support Scale was assessed [28]. This scale consists of 10 items indicating how helpful a person’s social support network is (e.g. *“If I want to talk about a problem*,* I know to whom I can go”)*. The scale is answered on a five-point Likert scale from 1 = “not true” to 5 = “very true”. A sum score of all 10 items was calculated. Internal consistencies are as follows: HC: *α* = 0.93; MDD: *α* = 0.91.

The s-IAT, BSMAS and FERUS were administered at baseline.

We explored severity of depressive symptoms using the Beck Depression Inventory (German version, BDI-II; [7]. It is a self-assessment scale comprising 21 items. For each item ratings between 0 (not at all) and 3 (very intensive) are given depending on the symptom complaint. Thus, BDI-II scores range from 0 to 63. Internal consistency was good in both groups.

The BDI-II was administered at both points of measurement.

### Methylation analysis

Methylation data was assessed at two measurement points. The first measurement point was at the beginning of the hospital stay and the second measurement point was two weeks later. Purification of genomic DNA was conducted automatically by means of a MagNA Pure^®^ LC system using a commercial extraction kit (MagNA Pure LC DNA isolation kit; Roche Diagnostics, Mannheim, Germany). Methylation was quantified by SEQ-IT GmbH & Co. KG (Kaiserslautern, Germany) using the Sequenom Epityper MassArray System (San Diego, CA, USA). All steps of the EpiTYPER assay were performed under routine conditions: After bisulfite treatment (EZ DNA Methylation-Lightning Kit (CE-IVD); Zymo research, Freiburg, Germany) of genomic DNA from peripheral blood, amplicons for the CpG-rich regions in the SLC6A4, NR3C1 and OXT promoter were designed with Agena’s EpiDESIGNER software (OXT: chr20:3071425–3071795; NR3C1: amplicon 1: chr5:143403938–143404340; amplicon 2: chr5:143403936–143404097; SLC6A4: chr17:30235345–30236068; amplicon 1: chr17:30235345–30235765; amplicon 2: chr17:30235734–30236068; hg38).

Following PCR amplification, RNA was transcribed in-vitro and subsequently base-specific cleavage using RNase A was performed. This resulted in fragmented RNA molecules of identical length differing in their nucleotide composition due to bisulfite treatment. After sample preparation, probes were processed on a MALDI-TOF platform (Agena; MassArray 4). Resulting data was preprocessed with the EpiTYPER Analyser and methylation status was quantified by analyzing the mass spectra.

### mRNA analysis

Expression analysis was performed for the first measurement point (t0) by SEQ-IT GmbH & Co. KG (Kaiserslautern, Germany). After manual extraction of RNA in accordance with manufacturer’s instructions using the PreAnalytiX^®^ PAXgene^®^ Blood RNA Kit (IVD) (PreAnalytiX, Hombrechtikon, Switzerland; for details regarding the extraction of RNA from whole blood, please refer to information provided by the manufacturer: https://www.preanalytix.com/products/blood/rna-/-mirna/paxgene-blood-rna-kit-ivd-fda/US), quantity and quality of extracted RNA samples was determined photometrically using the BioTek Epoch Microplate Spectrophotometer (Agilent, Santa Clara, CA, USA). Then, quantity was adjusted to 50 ng/µl. To remove DNA and prevent RNA degradation, extracted probes were treated according to manufacturer’s instruction with DNase I as well as the recombinant ribonuclease-inhibitor RNaseOUT (Invitrogen, Thermo Fisher Scientific, Waltham, MA, USA). Reverse transcription from RNA to cDNA was performed using SuperScript™ II reverse transcriptase in combination with Random-Hexamere-Primers (Invitrogen, Thermo Fisher Scientific, Waltham, MA, USA). Absolute RNA mass was 650 ng (13 µl à 50 ng/µl). Primers and probes for expression analysis (qPCR) were from TIB MOLBIOL (TIB MOLBIOL Syntheselabor GmbH, Berlin, Germany).

The selection of housekeeping genes was based on Vandesompele et al. [70]. qPCR was performed in duplicates using Hot Start Taq Probe qPCR Mix (ROX) (Axon Labortechnik GmbH, Kaiserslautern, Germany). Measurement was performed in duplicates using a LightCycler^®^ 480 Instrument II (Roche Diagnostics International AG, Rotkreuz, Switzerland). Cq values were obtained using the Second Derivative Maximum Method with Gene Maximization Set-up using two Inter-Run Calibrators [25]. Analysis of measures was performed by means of qbase + in accordance with Hellemans et al. [25].

### Blood cell count

Measurements of hematology were performed at Universitätsklinikum Ulm using a Sysmex XN-9100/Sysmex XN-1000 analyzer (Sysmex Corporation, Kobe, Japan) for both measurement points. Fluorescence flow cytometry employing fluorescent dyes in combination with semiconductor laser technology was used to determine cellular differentiation, enabling leukocyte differentiation. Relative abundance was provided by the Sysmex-Middleware Extended IPU (Sysmex Corporation, Kobe, Japan). Absolute abundance of leukocytes was calculated from relative measures of different leukocyte types and is given in Giga/l. In the interest of model parsimony, we used absolute abundance of lymphocytes and neutrophilic granulocytes as covariates in our analyses with group as factor. These two cell types are the most frequent nucleated cells in peripheral blood.

### Statistical analysis

Data were analyzed using R statistics (v. 4.2.2; (R Development Core Team, 2008) with the packages *MatchIt* [26], *psych* [58], *lme4* [6], *reshape* [74], *lmerTest* [32], *effects* [17], *sjplot* [35] and *ggplot2* [75]. First, data was matched using a propensity score approach with the nearest neighbor method for the variables age, sex and log-transformed alcohol consumption. Since patients smoked a significantly higher number of cigarettes per day and had a significantly higher body mass index (BMI), we could not match groups with respect to nicotine consumption or BMI. Instead, we included these variables as covariates in the LMMs and excluded all CpG sites associated with nicotine and BMI from correlation analyses (only one CpG unit was excluded due to this criterion: NR3C1 CpG 20.21, which was significantly associated with nicotine consumption in the MDD group, *r* = 0.40, *p*_*BH*_ = 0.011). We explored changes in depression severity between groups over time by performing a mixed ANOVA.

Welch’s t-tests were performed for group comparisons regarding the investigated variables (Table 2). Thereafter, for OXT 13 CpG units, for SLC6A4 27 CpG units and for NR3C1 16 CpG units were analyzed for both measurement points.

Exploratory Spearman’s correlation coefficients between s-IAT, BSMAS, FERUS and methylation were calculated for each group separately. Next, we fitted nine linear mixed effects models (three for each gene) with methylation status as dependent variable and problematic social media use or quality of social relationships (s-IAT total/Social Media Addiction Scale total/FERUS total; one model each), group and time as well as the interaction of problematic social media use/quality of social relationships and group and the interaction of problematic social media use/quality of social relationships and time as fixed effects. We allowed for random intercepts of participant and CpG-site. We also included baseline cell type composition measured as absolute abundance of lymphocytes and neutrophilic granulocytes, BDI baseline values, baseline values of dose equivalents for antidepressants and nicotine consumption at baseline as covariates. The first model was: methylation ~ neut_abs + lymph_abs + BDI + dose-equivalents for antidepressants + BMI + Nicotine +s-IAT total*group*time + (1|participant) + (1|CpG-site). Dose equivalents of neuroleptics were not included in the interest of model parsimony. Since the inclusion of the three-way interaction term between social media/internet use tendencies/quality of social relationships, group and time produced a rank-deficient fixed effects design matrix, we calculated difference values for methylation (t1-t0) and used methylation differences as dependent variables. The interaction between group and social media/internet use tendencies/quality of social relationships on methylation differences is equivalent to the repeated-measures approach with the three-way interaction. Next, we used a bootstrapping approach with 5000 iterations for each of the models to obtain the empirical densities for the model parameters.

## Results

### Group comparisons

There was a significant interaction of group and time in the mixed ANOVA with depression severity as dependent variable (MDD: t0: *M* = 29.11, *SD* = 12.70, t1: *M* = 21.10, *SD* = 13.11, HC: t0: *M* = 5.27, *SD* = 5.24, t1: *M* = 2.10, *SD* = 3.01; *F*(1,135) = 13.72, *p* < 0.001, *η*^*2*^ = 0.01). We found significant group differences between MDD and HC regarding the self-reported size and quality of their social network and regarding hours spent on social media per week. Group differences regarding problematic social media use and craving were not significant (Table 2). Group comparisons for all investigated variables are in Table S1.

**Table 2:** Descriptive statistics and group differences for the investigated variables

	HC			MDD						
Variable	*N*(m/f)			*N*(m/f)						
Sex	35/48			35/48						
	*Mean*	*SD*	*n*	*Mean*	*SD*	*n*	*t*	*df*	*p*_*BH*_	*d*
Age	40.25	13.28	83	42.23	14.11	83	-0.93	163.40	0.386	0.14
Nicotine	1.43	4.23	83	6.04	8.19	83	-4.56	122.86	0.000	0.71
Alcohol	4.11	6.76	83	9.78	48.62	83	-1.05	85.17	0.359	0.16
BMI	25.25	4.22	83	27.22	6.89	83	-2.22	135.95	0.067	0.35
Social network	3.28	0.72	83	1.90	0.72	72	11.89	150.25	0.000	-1.91
Internet use in h/week	22.21	19.41	83	27.74	28.61	57	-1.27	90.81	0.309	0.24
Social media use in h/week	8.52	7.08	77	18.10	27.29	58	-2.61	62.80	0.033	0.51
FERUS	44.04	7.43	83	35.77	11.09	82	5.62	141.34	0.000	-0.88
s-IAT total	21.72	8.20	83	23.12	8.97	81	-1.04	159.91	0.359	0.16
s-IAT loss of control	12.81	4.91	83	12.94	5.13	81	-0.17	161.22	0.867	0.03
s-IAT craving	8.92	3.71	83	10.19	4.37	81	-2.00	156.55	0.094	0.31
BSMAS	9.49	4.30	77	10.71	5.07	77	-1.61	148.02	0.187	0.26
DE antidepressants				59.50	43.10	74				
DE neuroleptics				25.65	58.77	77				

Note. HC: healthy controls; MDD: patients suffering from Major Depressive Disorder; s-IAT: short internet addiction test with the subscales loss of control and craving; BSMAS: Bergen Social Media Addiction Scale; DE: dose equivalents. *P*-values are adjusted for false discovery rate

### Correlation analyses

We found a significant positive association between problematic social media use (*r* = 0.36, *p* = 0.021, FDR corrected), IUD tendencies in general (*r* = 0.33, *p* = 0.024, FDR corrected) and specifically craving (*r* = 0.40, *p* = 0.004, FDR corrected) and NR3C1 CpG 9 methylation (chr5:143404042–143404043) in MDD. In addition, in healthy controls, we found a significant association between social support and OXT CpG 3 (chr20:3071469–3071470) methylation (*r* = 0.30, *p* = 0.048, FDR corrected). It is, however, worth mentioning that the vast majority of CpG sites in NR3C1 as well as OXT showed non-significant correlations (after FDR adjustment) with IUD tendencies or social media addiction tendencies in both groups.

We did not find significant associations between methylation of SLC6A4 and IUD/social media addiction tendencies after FDR correction. We did not find significant associations between mRNA abundance and IUD/social media addiction tendencies. We found two significant association between DNA methylation of SLC6A4 CpG 3 (chr17: 30235998–30235999) as well as CpG unit 5.6 (chr17: 30235972–30235975) and mRNA abundance of SLC6A4 in MDD (*r* = 0.46, *p* = 0.006 and *r* = 0.47, *p* = 0.006 respectively, FDR corrected) (for a complete table of correlation coefficients ordered by group and p-values, see Tables S2-S4).

### Linear mixed effects models

**Fig. 1:**
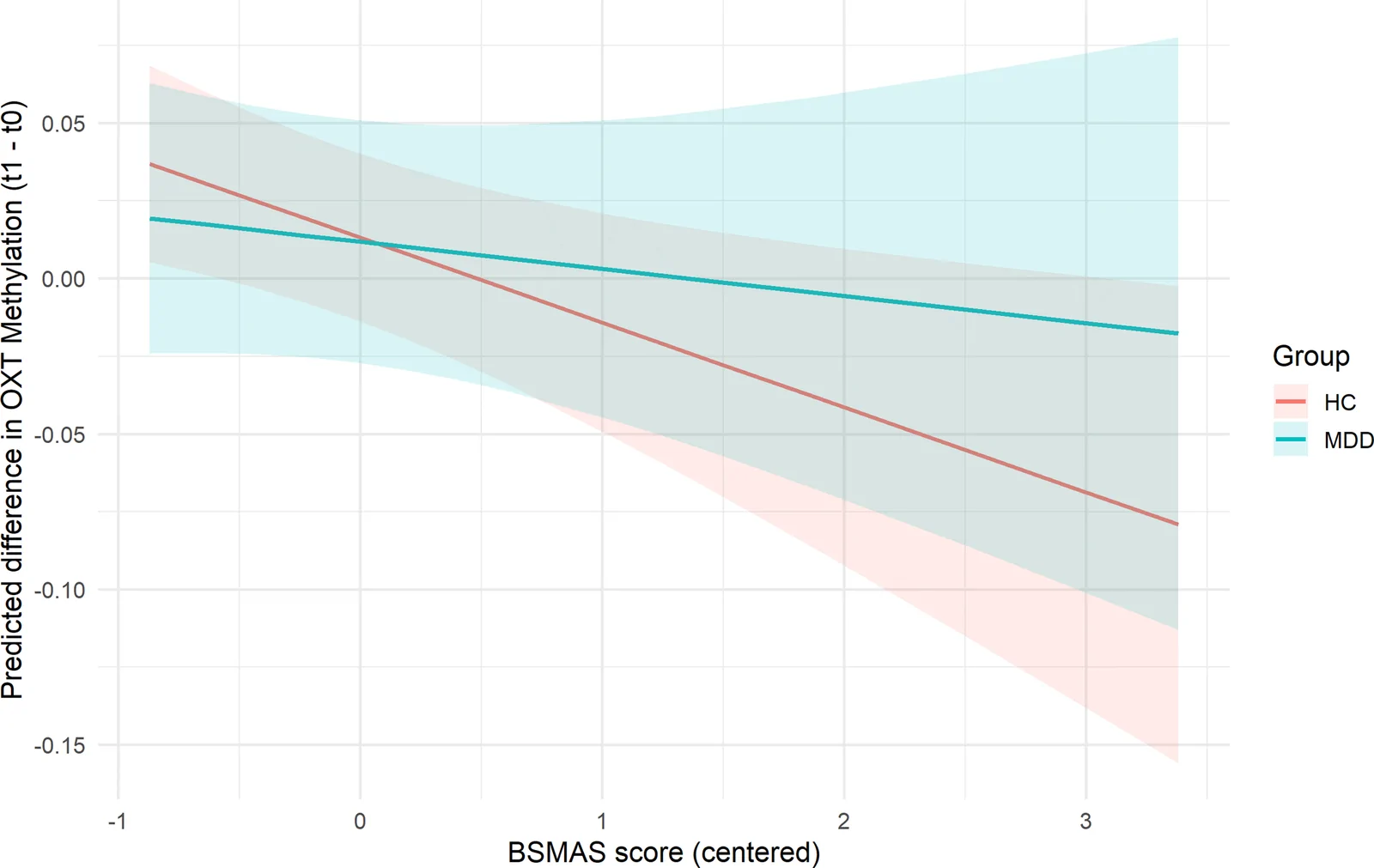
Effect of social media addiction tendencies on difference in OXT methylation (t1-t0) by group

Problematic Social media use tendencies were a significant predictor of the differences in OXT methylation (t1-t0). However, after bootstrapping, the confidence interval of this regression coefficient contained zero. Therefore, this effect should be interpreted with caution. The corresponding model is shown in Table 3, the effect of problematic social media use is shown in Fig. 1. Higher social media addiction tendencies were associated with decreasing differences in OXT methylation. This effect was independent of group. While fixed effects explained only a small amount of variance in differences of OXT methylation between measurement points (marginal *R*^*2*^ = 0.06), both fixed and random effects explained a medium amount of variance in the dependent variable (conditional *R*^*2*^ = 0.34).

All bootstrapped CIs of the remaining eight models can be found in the supplementary material (Tables S5-S12).

**Table 3:** Predictors for differences in OXT methylation, estimates and bootstrapped confidence intervals

Predictors	*β*	*CI*	*p*
(Intercept)	0.0097	-0.0433–0.0703	0.542
NG	0.0110	-0.0212–0.0445	0.206
Lymph	-0.0089	-0.0386–0.0253	0.311
BDI-II	-0.0092	-0.0548–0.0447	0.488
AD	-0.0080	-0.0426–0.0307	0.440
BMI	-0.0064	-0.0396–0.0262	0.480
Nicotine	-0.0025	-0.0429–0.0319	0.808
BSMAS	-0.0271	-0.0698–0.0126	0.009
Group	-0.0012	-0.1098–0.0975	0.968
BSMAS × Group	0.0186	-0.0367–0.0753	0.245

Note. BMI: Body Mass Index; BDI-II: baseline depression severity; AD: baseline dose equivalents of antidepressants; NG: neutrophilic granulocytes; Lymph: lymphocytes; BSMAS: Bergen Social Media Addiction Scale sum score; reference level for group was MDD

## Discussion

The present study is among the first to choose an epigenetic approach to study individual differences in unspecified IUD tendencies. Interestingly, we observed positive associations between one CpG site in NR3C1 (chr5:143404042–143404043) and problematic social media use as well as IUD tendencies in MDD. OXT methylation was associated with social support in HC. Furthermore, higher problematic social media use predicted smaller methylation changes in OXT in both groups. However, effect sizes in the LMM were very small and the CI contained zero. As for SLC6A4, methylation of two CpG sites was significantly associated with mRNA abundance and there were no significant associations between SLC6A4 methylation and problematic social media use/IUD tendencies. The latter is in line with heterogeneous findings on the role of the serotonin transporter polymorphism in IUDs [13, 33, 80]. Of note we are not the first group implicating epigenetics of SLC6A4 in IUDs: a recent work observed associations with higher and lower methylation patterns in IUD depending on the sites investigated and the cut-off scores used to investigate subgroups [5]. In this article, the authors report that one of six CpG sites (these six CpG sites 1–6 correspond with SLC6A4 CpG sites 5–10 in our analyses – assuming the authors count CpG sites from 3’ to 5’ as they show in Annunzi et al. [5] shows significantly higher methylation in the group having mild level of internet addiction tendencies as compared to the group without addiction/the group with more severe addiction tendencies. They also report lower levels of methylation in two different CpG sites in the group with mild addiction tendencies as compared to the group without addiction/the group with more severe addiction tendencies. Similarly, Annunzi et al. [5] report one positive (CpG 1 in their article; in our analyses –HC: CpG 5.6 and sIAT total: *r* = 0.12; MDD: CpG 5.6 and sIAT total: *r* = 0.00) and three negative (CpG 2, 3 and 5 in their article, in our analyses – HC: CpG 7 and sIAT total: *r* = -0.07; MDD: CpG 7 and sIAT total: *r* = 0.02) correlation coefficients between IAT score and SLC6A4 methylation. However, since we investigated a higher number of CpG sites and controlled FDR, our results concerning the associations between CpG sites investigated by Annunzi et al. [5] and IUD are non-significant. Another explanation for our non-significant findings considering SLC6A4 methylation is that this gene might be heavily influenced by SSRI treatment in MDD and thus there might be pharmacological contamination of this genomic location. Therefore, even the significant associations of SLC6A4 CpG 3 as well as CpG 5.6 (chr17: 30235998–30235999, chr17: 30235972–30235975) methylation and mRNA abundance might be associated with medication rather than depression.

The observations that social support is positively associated with OXT CpG 3 (chr20:3071469–3071470) methylation and that higher problematic social media use went along with smaller changes in OXT methylation over the 14 day period could indicate that social connections (on- and offline) shape OXT methylation. These results are in line with oxytocin being implicated in human sociability and with the idea that the social environment programs OXT [21, 37]. Since, again, effects were small, CIs contained zero and we did not find associations with mRNA levels (of note: we only have mRNA levels for t0), results should be interpreted cautiously. Therefore, our results do not support our initial rationale that IUD tendencies/problematic social media use could be a relevant factor influencing therapy success by their association with (changes in) DNA-methylation. This interpretation of the data should be understood as preliminary due to our small sample size.

While we found significant associations between methylation of a single NR3C1 CpG site and IUD tendencies as well as problematic social media use, methylation of most NR3C1 CpG sites showed weak, non-significant associations with IUD tendencies or problematic social media use. Since substance use is a maladaptive compensatory strategy and is also associated with a bodily stress response [66], the notion that methylation of NR3C1, a gene implicated in the bodily stress response, is associated with substance use [56] seems straightforward. Interestingly, NR3C1 methylation has already been investigated in the context of substance use disorders. Recent studies showed that NR3C1 hypermethylation was associated with future self-reports of recent use and use duration of any substance [56] and that chronic alcohol use was associated with increased NR3C1 methylation [19]. However, there are also studies that did not find associations between NR3C1 methylation and cannabis use disorder [51]. Our results are in line with the heterogeneous findings of earlier studies on substance use: We found an association between IUD tendencies and NR3C1 methylation for only one CpG site. Methylation of this CpG site was not associated with mRNA abundance and could therefore not be functionally biologically relevant. Of course, there are factors other than DNA-methylation influencing mRNA transcription like histones or chromatin remodellers [29, 53]. Therefore, small effects of DNA-methylation on transcription could be overshadowed by these factors and by our limited sample size.

### Limitations

In addition, when interpreting our findings it should be kept in mind, that depression is associated with substance/behavioral addictions. However, it is of note that we have not investigated a sample of patients diagnosed with IUD (aside from gaming and gambling disorder also no official diagnosis exist) and the study is hampered by its self-report nature going along with usual biases such as lack of introspective skills or answering in a socially desirable fashion. In addition, we assessed IUD tendencies/problematic social media use as habitual use. Therefore, associations between these habitual variables and dynamic methylation change could be difficult to detect, since IUD tendencies are shaped by multiple genetic, environmental, and developmental influences, whereas treatment associated changes in DNA methylation may reflect short-term biological adaption. Further, while we assessed and controlled for several covariates, there are factors that might be relevant for methylation differences or changes that were not accounted for in our study like for example time of day or fasting state [4, 52]. Additionally, while the investigation of cooccurring changes in IUD tendencies and their potential epigenetic underpinnings would provide valuable insights in pathophysiological mechanisms underlying IUD and strong arguments for potential targets for treating IUD, we think that it is still valuable to report how habitual IUD tendencies are associated with changes in epigenetic signatures for several reasons: First, there are only few studies that investigated changes in epigenetics in psychiatric disorders and we provide longitudinal methylation-data of several target genes that are relevant to both depression and IUD [5, 19, 60, 63]. Second, there are relevant comorbidities of depression and substance use disorders as well as behavioral addictions [23, 27]. Comorbidities at the beginning of treatment could be relevant for depression development, course, treatment and associated changes of underlying biological markers of depression [77, 79]. Third, even though we cannot infer whether changes in methylation are accompanied by changes in IUD tendencies, we provide a more nuanced picture of the depressed sample of inpatients we investigated.

Another limitation is that the two-week observation period might be too short to detect methylation changes relevant to treatment effects. Even though we observed a significant reduction in depression severity, several patients did not improve. If methylation changes explain only a certain amount of variance in symptom change, a much larger sample size would be necessary to reliably detect this small effect. In addition, even though there are studies suggesting that psychiatric disorders show significant symptom change within the first 14 days of treatment [49], the possibility remains that methylation changes are not due to treatment effects but rather reflect reactions to the circumstances around hospitalization and the adaption to the new environmental conditions. Lastly, it needs also to be mentioned that we assessed methylation patterns in peripheral blood, where one might ask how this relates to methylation patterns in the brain. Regarding methylation patterns of SLC6A4, research suggests that SLC6A4’s methylation patterns are indeed related to brain serotonin synthesis [72], to brain function [18] and brain methylation patterns in amygdala tissue [59].

Nevertheless, we applied strong correction procedures and observed that methylation of one CpG unit of the NR3C1 gene was robustly and positively associated with problematic internet and social media use. Further interesting findings were made in the context of problematic social media use scores and decline of OXT methylation differences. Our findings should thus encourage other scientists to join the field of the molecular study of IUDs to better characterize the nature of IUDs, an approach that is still scant in the literature. Only few attempts have been made to characterize the molecular genetic nature of IUD and epigenetic approaches are so far as nearly non-existent (but see a recent study focusing on the DAT gene coding for the dopamine transporter [47]; this study is limited though by the very small sample and is not discussed further here). We believe that more research in this area will not only supplement insights derived from brain imaging research in the area [68], but could also be combined in the near future with brain imaging studies to get a fuller understanding of the “Internet addicted” brain (see also parallels with smartphone overuse in [42].

Concluding, the present work is among the first to show that the study of epigenetics of Internet Use Disorder – here with a focus on problematic social media use – can provide interesting insights into the molecular underpinnings of this potential new disorder. Such insights are of relevance in the ongoing debate if certain kind of online behaviors such as problematic social media use warrant an own official diagnosis. For this it is also important to understand similarities between the molecular basis of substance and non-substance use disorders. This said, this field is just at its beginning.

## Supplementary Information

Below is the link to the electronic supplementary material.

Supplementary Material 1

## Data Availability

The datasets generated and/or analysed during the current study are not publicly available due to the statement of our ethics committee (protection of patient data) but are available from the corresponding author on reasonable request.

## Abbreviations

IUD: Internet use disorder
MDD: Major Depressive Disorder
HC: Healthy Controls
SSRI: Selective serotonin reuptake inhibitors
SNRI: Serotonin-norepinephrine reuptake inhibitors
TCA: Tricyclic antidepressants
TeCA: Tetracyclic antidepressants
ECT: Electroconvulsive therapy
BLT: Bright light therapy
CBT: Cognitive behavioral therapy
s-IAT: Short internet addiction test with the subscales loss of control and craving
BSMAS: Bergen Social Media Addiction Scale
DE: Dose equivalents
BMI: Body Mass Index
BDI-II: Baseline depression severity
AD: Baseline dose equivalents of antidepressants
NG: Neutrophilic granulocytes
Lymph: Lymphocytes
