# Supplementary material for: Epigenetics of generalized internet use disorder including data on overuse of social media: a closer look at the SLC6A4, NR3C1 and OXT genes
Source: BMC Psychiatry. 2026 Oct 14;26:789. doi: 10.1186/s12888-026-08743-1 (PMC13670694; doi:10.1186/s12888-026-08743-1)
Supplement: Supplementary file 1 [file 12888_2026_8743_MOESM1_ESM.docx]

**Epigenetics of generalized Internet Use Disorder including data on overuse of social media: a closer look at the SLC6A4, NR3C1 and OXT genes**

Christian Montag^1,2,3*^, Markus Kiefer^4^, Bernhard J. Connemann^4^, Thomas Kammer^4^, & Simon Sanwald^4^

^1^Centre for Cognitive and Brain Sciences, Institute of Collaborative Innovation, University Macau, Macau SAR, China

^2^Department of Psychology, Faculty of Social Sciences, University of Macau, Macau SAR, China

^3^Department of Computer and Information Science, Faculty of Science and Technology, University of Macau, Macau SAR, China

^4^Department of Psychiatry and Psychotherapy III, Ulm University, Ulm, Germany

**Group comparisons**

Table S1 shows explorative group comparisons for all investigated variables.

**Table S1.** Descriptive statistics and exploratory group comparisons for all investigated variables for both measurement points with uncorrected p-values.

|  | **HC** |  |  | **MDD** |  |  |  |  |  |  |
| --- | --- | --- | --- | --- | --- | --- | --- | --- | --- | --- |
| **Variable** | *Mean* | *SD* | *n* | *Mean* | *SD* | *n* | *t* | *df* | *p* | *d* |
| Lymphocytes relative | 30.14 | 8.30 | 68 | 28.59 | 7.07 | 61 | 1.15 | 126.67 | 0.254 | -0.20 |
| neutrophilic Granulocytes relative | 58.55 | 8.62 | 68 | 60.44 | 7.52 | 61 | -1.33 | 126.91 | 0.187 | 0.23 |
| Basophiles relative | 0.64 | 0.24 | 68 | 0.70 | 0.33 | 61 | -1.06 | 109.67 | 0.291 | 0.19 |
| Eosinophiles relative | 2.49 | 1.99 | 68 | 2.57 | 1.76 | 61 | -0.26 | 126.98 | 0.792 | 0.05 |
| Monocytes relative | 8.18 | 2.03 | 68 | 7.70 | 2.09 | 61 | 1.31 | 124.54 | 0.191 | -0.23 |
| Lymphocytes relative t1 | 31.48 | 9.22 | 68 | 28.46 | 7.88 | 59 | 1.99 | 124.98 | 0.048 | -0.35 |
| neutrophilic Granulocytes relative t1 | 56.90 | 10.21 | 68 | 60.33 | 7.94 | 59 | -2.13 | 123.58 | 0.035 | 0.37 |
| Basophiles relative t1 | 0.68 | 0.27 | 68 | 0.75 | 0.36 | 59 | -1.30 | 106.11 | 0.196 | 0.24 |
| Eosinophiles relative t1 | 2.67 | 2.46 | 68 | 2.87 | 3.13 | 59 | -0.39 | 109.62 | 0.696 | 0.07 |
| Monocytes relativee t1 | 8.27 | 1.96 | 68 | 7.59 | 1.86 | 59 | 2.01 | 124.06 | 0.046 | -0.36 |
| N_CpG_1.2 | 0.05 | 0.02 | 75 | 0.05 | 0.01 | 71 | 0.42 | 125.74 | 0.678 | -0.06 |
| N_CpG_3.4.5 | 0.06 | 0.02 | 75 | 0.06 | 0.01 | 71 | -0.29 | 132.95 | 0.770 | 0.06 |
| N_CpG_9 | 0.10 | 0.10 | 75 | 0.09 | 0.07 | 66 | 0.30 | 136.15 | 0.763 | -0.05 |
| N_CpG_10.11 | 0.10 | 0.07 | 68 | 0.13 | 0.08 | 67 | -2.05 | 127.54 | 0.042 | 0.35 |
| N_CpG_12.13 | 0.03 | 0.02 | 74 | 0.02 | 0.02 | 69 | 0.29 | 139.70 | 0.774 | -0.06 |
| N_CpG_14 | 0.01 | 0.01 | 72 | 0.01 | 0.02 | 67 | -1.94 | 105.62 | 0.055 | 0.31 |
| N_CpG_17.18 | 0.02 | 0.02 | 74 | 0.01 | 0.01 | 68 | 1.74 | 112.76 | 0.085 | -0.26 |
| N_CpG_20.21 | 0.01 | 0.02 | 75 | 0.02 | 0.03 | 69 | -2.39 | 102.42 | 0.019 | 0.41 |
| N_CpG_26 | 0.04 | 0.03 | 75 | 0.03 | 0.02 | 71 | 0.44 | 141.88 | 0.664 | -0.08 |
| N_CpG_35 | 0.58 | 0.17 | 75 | 0.61 | 0.14 | 71 | -1.01 | 140.19 | 0.314 | 0.17 |
| N_CpG_36 | 0.03 | 0.03 | 75 | 0.04 | 0.07 | 71 | -1.52 | 100.12 | 0.132 | 0.26 |
| N_CpG_38.39 | 0.01 | 0.01 | 75 | 0.01 | 0.01 | 69 | -0.70 | 141.09 | 0.485 | 0.08 |
| N_rev_CpG_6.7 | 0.08 | 0.02 | 78 | 0.08 | 0.02 | 74 | -0.39 | 145.74 | 0.696 | 0.06 |
| N_rev_CpG_8 | 0.17 | 0.03 | 78 | 0.18 | 0.11 | 74 | -0.85 | 84.78 | 0.398 | 0.14 |
| N_rev_CpG_10.11 | 0.04 | 0.01 | 78 | 0.04 | 0.01 | 74 | 2.02 | 146.80 | 0.045 | -0.33 |
| N_rev_CpG_12.13 | 0.02 | 0.01 | 77 | 0.02 | 0.01 | 74 | -2.64 | 132.88 | 0.009 | 0.41 |
| N_CpG_1.2.t1 | 0.05 | 0.02 | 69 | 0.06 | 0.02 | 57 | -0.53 | 121.99 | 0.596 | 0.13 |
| N_CpG_3.4.5.t1 | 0.06 | 0.02 | 69 | 0.06 | 0.02 | 57 | -0.65 | 118.28 | 0.517 | 0.10 |
| N_CpG_9.t1 | 0.05 | 0.03 | 64 | 0.04 | 0.06 | 57 | 1.23 | 85.36 | 0.222 | -0.23 |
| N_CpG_10.11.t1 | 0.12 | 0.06 | 50 | 0.11 | 0.07 | 49 | 1.39 | 94.05 | 0.168 | -0.28 |
| N_CpG_12.13.t1 | 0.03 | 0.02 | 69 | 0.04 | 0.03 | 57 | -0.87 | 96.47 | 0.388 | 0.16 |
| N_CpG_14.t1 | 0.01 | 0.02 | 52 | 0.01 | 0.03 | 34 | -0.02 | 63.81 | 0.984 | 0.00 |
| N_CpG_17.18.t1 | 0.02 | 0.04 | 68 | 0.03 | 0.05 | 52 | -1.09 | 100.83 | 0.277 | 0.22 |
| N_CpG_20.21.t1 | 0.03 | 0.03 | 69 | 0.03 | 0.03 | 57 | -1.16 | 111.82 | 0.250 | 0.20 |
| N_CpG_26.t1 | 0.03 | 0.03 | 69 | 0.05 | 0.05 | 57 | -2.23 | 88.94 | 0.028 | 0.42 |
| N_CpG_35.t1 | 0.62 | 0.15 | 69 | 0.74 | 0.07 | 57 | -6.28 | 97.83 | 0.000 | 1.05 |
| N_CpG_36.t1 | 0.03 | 0.04 | 69 | 0.06 | 0.07 | 55 | -2.83 | 84.52 | 0.006 | 0.54 |
| N_CpG_38.39.t1 | 0.02 | 0.02 | 69 | 0.02 | 0.02 | 56 | -1.21 | 109.71 | 0.227 | 0.22 |
| N_rev_CpG_6.7.t1 | 0.08 | 0.02 | 77 | 0.07 | 0.01 | 63 | 4.24 | 123.65 | 0.000 | -0.66 |
| N_rev_CpG_8.t1 | 0.16 | 0.10 | 77 | 0.13 | 0.03 | 63 | 2.43 | 87.61 | 0.017 | -0.38 |
| N_rev_CpG_10.11.t1 | 0.03 | 0.01 | 77 | 0.03 | 0.01 | 63 | 0.15 | 119.56 | 0.881 | -0.09 |
| N_rev_CpG_12.13.t1 | 0.02 | 0.02 | 76 | 0.02 | 0.01 | 63 | -0.03 | 137.00 | 0.973 | 0.00 |
| mrnaNR3 | 1.25 | 0.45 | 67 | 0.96 | 0.42 | 53 | 3.70 | 114.50 | 0.000 | -0.68 |
| O_CpG_1.2 | 0.54 | 0.11 | 78 | 0.54 | 0.11 | 76 | -0.26 | 151.53 | 0.794 | 0.05 |
| O_CpG_3 | 0.54 | 0.10 | 78 | 0.54 | 0.11 | 76 | 0.05 | 151.14 | 0.961 | 0.00 |
| O_CpG_4 | 0.56 | 0.11 | 78 | 0.51 | 0.12 | 74 | 2.43 | 146.44 | 0.016 | -0.39 |
| O_CpG_5 | 0.73 | 0.13 | 69 | 0.69 | 0.12 | 66 | 2.00 | 132.90 | 0.047 | -0.35 |
| O_CpG_7.8 | 0.49 | 0.12 | 78 | 0.47 | 0.13 | 76 | 1.31 | 151.29 | 0.192 | -0.21 |
| O_CpG_9.10 | 0.37 | 0.10 | 78 | 0.36 | 0.10 | 75 | 1.13 | 150.75 | 0.259 | -0.19 |
| O_CpG_13 | 0.34 | 0.11 | 78 | 0.32 | 0.11 | 75 | 1.20 | 150.94 | 0.234 | -0.18 |
| O_CpG_14.15 | 0.38 | 0.10 | 78 | 0.36 | 0.12 | 76 | 0.89 | 146.22 | 0.374 | -0.15 |
| O_CpG_16 | 0.19 | 0.08 | 78 | 0.18 | 0.07 | 76 | 0.79 | 150.49 | 0.433 | -0.13 |
| O_CpG_18 | 0.48 | 0.13 | 69 | 0.50 | 0.12 | 63 | -0.57 | 129.47 | 0.568 | 0.10 |
| O_CpG_19 | 0.20 | 0.13 | 70 | 0.21 | 0.14 | 66 | -0.23 | 131.82 | 0.815 | 0.04 |
| O_CpG_24.25 | 0.19 | 0.09 | 67 | 0.22 | 0.12 | 65 | -1.50 | 118.70 | 0.135 | 0.27 |
| O_CpG_26 | 0.18 | 0.07 | 78 | 0.17 | 0.09 | 76 | 1.15 | 145.09 | 0.252 | -0.18 |
| O_CpG_1.2.t1 | 0.58 | 0.11 | 75 | 0.52 | 0.12 | 56 | 2.60 | 114.12 | 0.011 | -0.46 |
| O_CpG_3.t1 | 0.56 | 0.12 | 75 | 0.54 | 0.12 | 56 | 0.93 | 118.19 | 0.355 | -0.16 |
| O_CpG_4.t1 | 0.57 | 0.13 | 75 | 0.52 | 0.14 | 56 | 2.31 | 116.97 | 0.023 | -0.41 |
| O_CpG_5.t1 | 0.76 | 0.14 | 55 | 0.74 | 0.15 | 20 | 0.55 | 31.68 | 0.587 | -0.15 |
| O_CpG_7.8.t1 | 0.51 | 0.12 | 75 | 0.46 | 0.14 | 56 | 2.20 | 104.56 | 0.030 | -0.40 |
| O_CpG_9.10.t1 | 0.39 | 0.10 | 74 | 0.34 | 0.10 | 56 | 2.32 | 119.70 | 0.022 | -0.41 |
| O_CpG_13.t1 | 0.37 | 0.12 | 75 | 0.33 | 0.09 | 56 | 2.40 | 129.00 | 0.018 | -0.41 |
| O_CpG_14.15.t1 | 0.40 | 0.12 | 75 | 0.34 | 0.09 | 56 | 3.75 | 128.16 | 0.000 | -0.64 |
| O_CpG_16.t1 | 0.20 | 0.11 | 75 | 0.17 | 0.06 | 56 | 1.92 | 113.35 | 0.057 | -0.30 |
| O_CpG_18.t1 | 0.56 | 0.11 | 56 | 0.51 | 0.08 | 28 | 2.38 | 72.21 | 0.020 | -0.50 |
| O_CpG_19.t1 | 0.27 | 0.14 | 52 | 0.26 | 0.19 | 24 | 0.23 | 35.07 | 0.819 | -0.07 |
| O_CpG_24.25.t1 | 0.23 | 0.09 | 48 | 0.20 | 0.10 | 21 | 1.13 | 36.27 | 0.268 | -0.30 |
| O_CpG_26.t1 | 0.18 | 0.08 | 75 | 0.16 | 0.06 | 56 | 1.81 | 128.49 | 0.073 | -0.31 |
| mrnaOXT | 1.11 | 0.76 | 67 | 1.08 | 0.89 | 51 | 0.19 | 97.34 | 0.849 | -0.04 |
| S_CpG_1 | 0.04 | 0.03 | 78 | 0.04 | 0.03 | 77 | 0.71 | 148.00 | 0.481 | -0.13 |
| S_CpG_3 | 0.04 | 0.05 | 77 | 0.05 | 0.07 | 77 | -1.98 | 141.83 | 0.050 | 0.31 |
| S_CpG_4 | 0.02 | 0.03 | 77 | 0.02 | 0.03 | 77 | 0.49 | 149.69 | 0.627 | -0.07 |
| S_CpG_5.6 | 0.09 | 0.07 | 77 | 0.13 | 0.09 | 74 | -3.16 | 141.91 | 0.002 | 0.53 |
| S_CpG_7 | 0.14 | 0.13 | 63 | 0.06 | 0.07 | 57 | 3.88 | 100.58 | 0.000 | -0.69 |
| S_CpG_8.9 | 0.03 | 0.04 | 78 | 0.03 | 0.03 | 77 | -0.07 | 151.52 | 0.946 | 0.03 |
| S_CpG_10 | 0.04 | 0.04 | 77 | 0.05 | 0.05 | 75 | -1.26 | 140.43 | 0.211 | 0.21 |
| S_CpG_11-15 | 0.04 | 0.02 | 78 | 0.04 | 0.02 | 77 | 2.40 | 150.92 | 0.018 | -0.36 |
| S_CpG_16.17 | 0.05 | 0.02 | 77 | 0.05 | 0.03 | 76 | 0.02 | 142.61 | 0.984 | 0.00 |
| S_CpG_18.19 | 0.06 | 0.02 | 76 | 0.06 | 0.02 | 77 | 0.86 | 150.24 | 0.392 | -0.17 |
| S_CpG_28 | 0.03 | 0.02 | 78 | 0.03 | 0.02 | 77 | 1.20 | 151.92 | 0.231 | -0.19 |
| S_CpG_29.30 | 0.03 | 0.02 | 78 | 0.03 | 0.02 | 77 | -0.08 | 149.43 | 0.939 | 0.06 |
| S_CpG_31.32 | 0.01 | 0.01 | 69 | 0.01 | 0.02 | 68 | -0.49 | 113.05 | 0.629 | 0.06 |
| S_CpG_39-42 | 0.07 | 0.04 | 70 | 0.08 | 0.06 | 69 | -0.42 | 123.56 | 0.679 | 0.06 |
| S_CpG_45 | 0.15 | 0.04 | 70 | 0.16 | 0.05 | 69 | -1.40 | 119.63 | 0.164 | 0.24 |
| S_CpG_46 | 0.04 | 0.04 | 69 | 0.04 | 0.06 | 67 | -0.54 | 119.72 | 0.590 | 0.09 |
| S_CpG_47.48 | 0.21 | 0.04 | 70 | 0.21 | 0.05 | 69 | -0.09 | 127.74 | 0.926 | 0.00 |
| S_CpG_52 | 0.04 | 0.05 | 70 | 0.04 | 0.04 | 68 | -0.12 | 135.96 | 0.908 | 0.02 |
| S_CpG_53.54 | 0.04 | 0.03 | 68 | 0.05 | 0.04 | 59 | -0.90 | 92.97 | 0.372 | 0.14 |
| S_CpG_57.58 | 0.20 | 0.07 | 69 | 0.19 | 0.08 | 69 | 0.87 | 132.51 | 0.385 | -0.16 |
| S_CpG_60 | 0.14 | 0.08 | 69 | 0.15 | 0.12 | 68 | -0.52 | 119.31 | 0.606 | 0.09 |
| S_CpG_61-63 | 0.11 | 0.04 | 69 | 0.11 | 0.05 | 68 | -0.53 | 128.87 | 0.595 | 0.08 |
| S_CpG_64 | 0.20 | 0.07 | 70 | 0.22 | 0.09 | 69 | -1.33 | 132.19 | 0.186 | 0.22 |
| S_CpG_65 | 0.37 | 0.15 | 66 | 0.38 | 0.14 | 62 | -0.16 | 125.76 | 0.873 | 0.03 |
| S_CpG_66 | 0.16 | 0.09 | 70 | 0.17 | 0.10 | 68 | -0.51 | 133.74 | 0.610 | 0.09 |
| S_CpG_69 | 0.17 | 0.07 | 70 | 0.17 | 0.11 | 68 | -0.31 | 111.69 | 0.755 | 0.06 |
| S_CpG_70.71 | 0.32 | 0.11 | 70 | 0.31 | 0.10 | 66 | 0.65 | 133.95 | 0.517 | -0.11 |
| S_CpG_1.t1 | 0.04 | 0.03 | 76 | 0.04 | 0.03 | 61 | -1.24 | 128.30 | 0.219 | 0.22 |
| S_CpG_3.t1 | 0.06 | 0.05 | 71 | 0.06 | 0.06 | 56 | -0.36 | 113.47 | 0.719 | 0.06 |
| S_CpG_4.t1 | 0.05 | 0.07 | 76 | 0.06 | 0.13 | 61 | -0.94 | 88.36 | 0.348 | 0.17 |
| S_CpG_5.6.t1 | 0.08 | 0.06 | 70 | 0.09 | 0.07 | 58 | -0.72 | 114.27 | 0.476 | 0.13 |
| S_CpG_7.t1 | 0.12 | 0.13 | 29 | 0.08 | 0.09 | 21 | 1.29 | 47.89 | 0.204 | -0.35 |
| S_CpG_8.9.t1 | 0.03 | 0.02 | 76 | 0.04 | 0.03 | 61 | -0.98 | 101.20 | 0.329 | 0.17 |
| S_CpG_10.t1 | 0.03 | 0.03 | 75 | 0.05 | 0.03 | 60 | -2.53 | 124.00 | 0.013 | 0.44 |
| S_CpG_11-15.t1 | 0.03 | 0.02 | 76 | 0.04 | 0.02 | 60 | -1.77 | 132.46 | 0.080 | 0.30 |
| S_CpG_16.17.t1 | 0.06 | 0.03 | 76 | 0.07 | 0.03 | 61 | -1.76 | 130.99 | 0.080 | 0.29 |
| S_CpG_18.19.t1 | 0.06 | 0.05 | 75 | 0.06 | 0.02 | 61 | 0.13 | 115.07 | 0.900 | -0.03 |
| S_CpG_28.t1 | 0.04 | 0.03 | 76 | 0.04 | 0.02 | 61 | -0.65 | 134.98 | 0.519 | 0.07 |
| S_CpG_29.30.t1 | 0.03 | 0.02 | 76 | 0.03 | 0.02 | 61 | -0.94 | 134.83 | 0.348 | 0.15 |
| S_CpG_31.32.t1 | 0.01 | 0.02 | 73 | 0.02 | 0.03 | 47 | -2.11 | 72.34 | 0.038 | 0.43 |
| S_CpG_39-42.t1 | 0.08 | 0.06 | 73 | 0.08 | 0.04 | 38 | -0.36 | 104.25 | 0.724 | 0.06 |
| S_CpG_45.t1 | 0.16 | 0.05 | 73 | 0.16 | 0.04 | 47 | -0.56 | 109.37 | 0.576 | 0.10 |
| S_CpG_46.t1 | 0.05 | 0.06 | 70 | 0.04 | 0.07 | 35 | 0.46 | 58.55 | 0.651 | -0.11 |
| S_CpG_47.48.t1 | 0.21 | 0.04 | 73 | 0.23 | 0.05 | 47 | -2.42 | 89.91 | 0.017 | 0.46 |
| S_CpG_52.t1 | 0.04 | 0.05 | 73 | 0.04 | 0.03 | 46 | 0.27 | 116.67 | 0.786 | -0.05 |
| S_CpG_53.54.t1 | 0.04 | 0.06 | 65 | 0.04 | 0.03 | 35 | 0.20 | 95.57 | 0.840 | -0.04 |
| S_CpG_57.58.t1 | 0.20 | 0.10 | 73 | 0.20 | 0.07 | 45 | -0.57 | 113.65 | 0.572 | 0.10 |
| S_CpG_60.t1 | 0.13 | 0.12 | 73 | 0.14 | 0.09 | 47 | -0.49 | 115.85 | 0.622 | 0.09 |
| S_CpG_61-63.t1 | 0.10 | 0.06 | 73 | 0.11 | 0.05 | 45 | -0.10 | 109.27 | 0.925 | 0.02 |
| S_CpG_64.t1 | 0.20 | 0.10 | 73 | 0.21 | 0.09 | 42 | -0.88 | 89.46 | 0.379 | 0.18 |
| S_CpG_65.t1 | 0.46 | 0.13 | 69 | 0.51 | 0.12 | 32 | -1.66 | 65.99 | 0.102 | 0.34 |
| S_CpG_66.t1 | 0.21 | 0.13 | 73 | 0.22 | 0.14 | 44 | -0.67 | 83.19 | 0.507 | 0.13 |
| S_CpG_69.t1 | 0.21 | 0.12 | 73 | 0.19 | 0.12 | 47 | 0.76 | 102.85 | 0.448 | -0.14 |
| S_CpG_70.71.t1 | 0.30 | 0.11 | 72 | 0.31 | 0.11 | 47 | -0.24 | 99.41 | 0.808 | 0.04 |
| mrnaSLC | 1.06 | 0.73 | 67 | 1.21 | 0.79 | 53 | -1.03 | 106.93 | 0.304 | 0.19 |

Note. Variables ending on t1 represent the second measurement point.

**Correlation coefficients**

Table S2-S4 show all associations between IUD tendencies, social support network and single CpG sites as well as mRNA abundance of the investigated genes for HC and MDD.

**Table S2.** Spearman correlation coefficients between methylation and IUD tendencies, social support and mRNA abundance for NR3C1

|  |  | HC | | | MDD | | |
| --- | --- | --- | --- | --- | --- | --- | --- |
| CpG-unit | Variable | rho | p | pBH | rho | p | pBH |
| N_CpG_1.2 | FERUS_SUM | -0,13 | 0,282 | 0,891 | -0,08 | 0,526 | 0,681 |
| N_CpG_1.2 | mrnaNR3 | 0,06 | 0,642 | 0,833 | 0,07 | 0,617 | 0,786 |
| N_CpG_1.2 | siat_craving | 0,10 | 0,414 | 0,657 | -0,27 | 0,022 | 0,070 |
| N_CpG_1.2 | siat_loc | 0,17 | 0,154 | 0,485 | -0,13 | 0,282 | 0,446 |
| N_CpG_1.2 | siat_total | 0,15 | 0,202 | 0,584 | -0,20 | 0,106 | 0,232 |
| N_CpG_1.2 | socm_sum | 0,16 | 0,186 | 0,511 | -0,02 | 0,899 | 0,963 |
| N_CpG_10.11 | FERUS_SUM | 0,04 | 0,722 | 0,891 | -0,09 | 0,470 | 0,646 |
| N_CpG_10.11 | mrnaNR3 | 0,01 | 0,956 | 0,956 | -0,04 | 0,794 | 0,867 |
| N_CpG_10.11 | siat_craving | 0,00 | 0,969 | 0,969 | -0,02 | 0,870 | 0,957 |
| N_CpG_10.11 | siat_loc | -0,02 | 0,895 | 0,984 | -0,02 | 0,847 | 0,888 |
| N_CpG_10.11 | siat_total | -0,02 | 0,853 | 0,912 | -0,05 | 0,695 | 0,740 |
| N_CpG_10.11 | socm_sum | -0,04 | 0,722 | 0,836 | 0,03 | 0,811 | 0,963 |
| N_CpG_12.13 | FERUS_SUM | -0,07 | 0,541 | 0,891 | 0,03 | 0,805 | 0,843 |
| N_CpG_12.13 | mrnaNR3 | -0,10 | 0,421 | 0,833 | 0,15 | 0,322 | 0,709 |
| N_CpG_12.13 | siat_craving | -0,17 | 0,155 | 0,570 | -0,05 | 0,699 | 0,809 |
| N_CpG_12.13 | siat_loc | -0,13 | 0,283 | 0,622 | 0,01 | 0,928 | 0,928 |
| N_CpG_12.13 | siat_total | -0,15 | 0,212 | 0,584 | -0,01 | 0,906 | 0,906 |
| N_CpG_12.13 | socm_sum | -0,17 | 0,168 | 0,511 | -0,30 | 0,017 | 0,064 |
| N_CpG_14 | FERUS_SUM | 0,06 | 0,628 | 0,891 | 0,13 | 0,308 | 0,557 |
| N_CpG_14 | mrnaNR3 | -0,09 | 0,479 | 0,833 | -0,04 | 0,800 | 0,867 |
| N_CpG_14 | siat_craving | -0,08 | 0,508 | 0,657 | -0,28 | 0,025 | 0,070 |
| N_CpG_14 | siat_loc | 0,00 | 0,984 | 0,984 | -0,13 | 0,284 | 0,446 |
| N_CpG_14 | siat_total | -0,02 | 0,871 | 0,912 | -0,18 | 0,160 | 0,320 |
| N_CpG_14 | socm_sum | -0,05 | 0,680 | 0,836 | -0,23 | 0,071 | 0,172 |
| N_CpG_17.18 | FERUS_SUM | -0,18 | 0,132 | 0,891 | -0,17 | 0,176 | 0,440 |
| N_CpG_17.18 | mrnaNR3 | -0,08 | 0,543 | 0,833 | 0,20 | 0,181 | 0,569 |
| N_CpG_17.18 | siat_craving | -0,09 | 0,422 | 0,657 | 0,18 | 0,150 | 0,235 |
| N_CpG_17.18 | siat_loc | 0,09 | 0,457 | 0,714 | 0,29 | 0,017 | 0,073 |
| N_CpG_17.18 | siat_total | 0,03 | 0,826 | 0,912 | 0,23 | 0,056 | 0,138 |
| N_CpG_17.18 | socm_sum | -0,08 | 0,485 | 0,836 | 0,23 | 0,060 | 0,164 |
| N_CpG_26 | FERUS_SUM | 0,24 | 0,040 | 0,439 | 0,00 | 0,983 | 0,983 |
| N_CpG_26 | mrnaNR3 | -0,01 | 0,943 | 0,956 | -0,07 | 0,640 | 0,786 |
| N_CpG_26 | siat_craving | 0,04 | 0,702 | 0,813 | -0,17 | 0,148 | 0,235 |
| N_CpG_26 | siat_loc | 0,02 | 0,832 | 0,984 | -0,15 | 0,219 | 0,437 |
| N_CpG_26 | siat_total | 0,03 | 0,812 | 0,912 | -0,15 | 0,211 | 0,357 |
| N_CpG_26 | socm_sum | -0,06 | 0,637 | 0,836 | -0,17 | 0,172 | 0,345 |
| N_CpG_3.4.5 | FERUS_SUM | -0,12 | 0,319 | 0,891 | -0,12 | 0,334 | 0,557 |
| N_CpG_3.4.5 | mrnaNR3 | 0,07 | 0,578 | 0,833 | 0,18 | 0,216 | 0,595 |
| N_CpG_3.4.5 | siat_craving | 0,10 | 0,393 | 0,657 | -0,20 | 0,090 | 0,199 |
| N_CpG_3.4.5 | siat_loc | 0,18 | 0,115 | 0,421 | -0,09 | 0,477 | 0,616 |
| N_CpG_3.4.5 | siat_total | 0,16 | 0,168 | 0,584 | -0,14 | 0,259 | 0,380 |
| N_CpG_3.4.5 | socm_sum | 0,13 | 0,277 | 0,678 | 0,06 | 0,607 | 0,888 |
| N_CpG_35 | FERUS_SUM | -0,03 | 0,819 | 0,892 | -0,05 | 0,670 | 0,737 |
| N_CpG_35 | mrnaNR3 | 0,27 | 0,031 | 0,282 | 0,10 | 0,503 | 0,786 |
| N_CpG_35 | siat_craving | -0,28 | 0,016 | 0,072 | -0,07 | 0,584 | 0,714 |
| N_CpG_35 | siat_loc | -0,26 | 0,023 | 0,100 | -0,10 | 0,388 | 0,570 |
| N_CpG_35 | siat_total | -0,28 | 0,015 | 0,065 | -0,09 | 0,448 | 0,553 |
| N_CpG_35 | socm_sum | -0,24 | 0,041 | 0,157 | -0,08 | 0,504 | 0,853 |
| N_CpG_36 | FERUS_SUM | 0,03 | 0,792 | 0,892 | 0,11 | 0,371 | 0,557 |
| N_CpG_36 | mrnaNR3 | 0,19 | 0,130 | 0,714 | -0,22 | 0,131 | 0,493 |
| N_CpG_36 | siat_craving | 0,09 | 0,422 | 0,657 | -0,19 | 0,111 | 0,223 |
| N_CpG_36 | siat_loc | 0,00 | 0,979 | 0,984 | -0,14 | 0,253 | 0,446 |
| N_CpG_36 | siat_total | 0,03 | 0,771 | 0,912 | -0,15 | 0,227 | 0,357 |
| N_CpG_36 | socm_sum | -0,06 | 0,617 | 0,836 | -0,06 | 0,646 | 0,888 |
| N_CpG_38.39 | FERUS_SUM | -0,06 | 0,608 | 0,891 | -0,19 | 0,122 | 0,396 |
| N_CpG_38.39 | mrnaNR3 | -0,03 | 0,842 | 0,926 | -0,10 | 0,480 | 0,786 |
| N_CpG_38.39 | siat_craving | -0,14 | 0,227 | 0,639 | -0,08 | 0,493 | 0,638 |
| N_CpG_38.39 | siat_loc | 0,08 | 0,487 | 0,714 | -0,09 | 0,469 | 0,616 |
| N_CpG_38.39 | siat_total | 0,01 | 0,917 | 0,917 | -0,11 | 0,384 | 0,528 |
| N_CpG_38.39 | socm_sum | -0,02 | 0,853 | 0,894 | 0,01 | 0,927 | 0,963 |
| N_CpG_9 | FERUS_SUM | 0,15 | 0,195 | 0,891 | -0,20 | 0,100 | 0,396 |
| N_CpG_9 | mrnaNR3 | -0,06 | 0,651 | 0,833 | 0,17 | 0,253 | 0,619 |
| N_CpG_9 | siat_craving | -0,14 | 0,232 | 0,639 | 0,40 | 0,001 | 0,004 |
| N_CpG_9 | siat_loc | -0,13 | 0,250 | 0,622 | 0,26 | 0,040 | 0,110 |
| N_CpG_9 | siat_total | -0,13 | 0,250 | 0,612 | 0,33 | 0,007 | 0,024 |
| N_CpG_9 | socm_sum | -0,06 | 0,593 | 0,836 | 0,36 | 0,005 | 0,021 |
| N_rev_CpG_10.11 | FERUS_SUM | 0,01 | 0,931 | 0,931 | -0,16 | 0,180 | 0,440 |
| N_rev_CpG_10.11 | mrnaNR3 | -0,13 | 0,283 | 0,833 | -0,07 | 0,643 | 0,786 |
| N_rev_CpG_10.11 | siat_craving | -0,10 | 0,373 | 0,657 | 0,11 | 0,358 | 0,525 |
| N_rev_CpG_10.11 | siat_loc | -0,12 | 0,282 | 0,622 | 0,15 | 0,209 | 0,437 |
| N_rev_CpG_10.11 | siat_total | -0,12 | 0,281 | 0,619 | 0,16 | 0,179 | 0,329 |
| N_rev_CpG_10.11 | socm_sum | -0,24 | 0,043 | 0,157 | 0,06 | 0,637 | 0,888 |
| N_rev_CpG_12.13 | FERUS_SUM | 0,04 | 0,729 | 0,891 | -0,06 | 0,603 | 0,722 |
| N_rev_CpG_12.13 | mrnaNR3 | -0,10 | 0,451 | 0,833 | -0,31 | 0,025 | 0,183 |
| N_rev_CpG_12.13 | siat_craving | 0,05 | 0,638 | 0,779 | -0,09 | 0,426 | 0,586 |
| N_rev_CpG_12.13 | siat_loc | 0,02 | 0,860 | 0,984 | -0,07 | 0,532 | 0,616 |
| N_rev_CpG_12.13 | siat_total | 0,04 | 0,738 | 0,912 | -0,09 | 0,452 | 0,553 |
| N_rev_CpG_12.13 | socm_sum | 0,08 | 0,486 | 0,836 | -0,12 | 0,316 | 0,580 |
| N_rev_CpG_6.7 | FERUS_SUM | 0,09 | 0,448 | 0,891 | -0,06 | 0,623 | 0,722 |
| N_rev_CpG_6.7 | mrnaNR3 | 0,26 | 0,038 | 0,282 | -0,10 | 0,476 | 0,786 |
| N_rev_CpG_6.7 | siat_craving | -0,01 | 0,948 | 0,969 | 0,00 | 0,979 | 0,979 |
| N_rev_CpG_6.7 | siat_loc | 0,07 | 0,538 | 0,740 | -0,08 | 0,511 | 0,616 |
| N_rev_CpG_6.7 | siat_total | 0,05 | 0,651 | 0,912 | -0,06 | 0,591 | 0,685 |
| N_rev_CpG_6.7 | socm_sum | -0,01 | 0,949 | 0,949 | 0,04 | 0,737 | 0,953 |
| N_rev_CpG_8 | FERUS_SUM | 0,10 | 0,371 | 0,891 | -0,15 | 0,212 | 0,467 |
| N_rev_CpG_8 | mrnaNR3 | -0,14 | 0,258 | 0,833 | -0,08 | 0,585 | 0,786 |
| N_rev_CpG_8 | siat_craving | -0,08 | 0,502 | 0,657 | 0,22 | 0,062 | 0,153 |
| N_rev_CpG_8 | siat_loc | -0,05 | 0,670 | 0,867 | 0,21 | 0,080 | 0,195 |
| N_rev_CpG_8 | siat_total | -0,07 | 0,565 | 0,888 | 0,22 | 0,057 | 0,138 |
| N_rev_CpG_8 | socm_sum | -0,07 | 0,577 | 0,836 | 0,27 | 0,027 | 0,086 |
| mrnaNR3 | FERUS_SUM | 0,08 | 0,523 | 0,891 | 0,12 | 0,380 | 0,557 |
| mrnaNR3 | siat_craving | 0,09 | 0,490 | 0,657 | 0,21 | 0,134 | 0,235 |
| mrnaNR3 | siat_loc | 0,11 | 0,364 | 0,714 | 0,31 | 0,025 | 0,078 |
| mrnaNR3 | siat_total | 0,11 | 0,394 | 0,789 | 0,28 | 0,045 | 0,138 |
| mrnaNR3 | socm_sum | 0,05 | 0,720 | 0,836 | 0,03 | 0,848 | 0,963 |

**Table S3.** Spearman correlation coefficients between methylation and IUD tendencies, social support and mRNA abundance for OXT

|  |  | HC | | | MDD | | |
| --- | --- | --- | --- | --- | --- | --- | --- |
| CpG-unit | Variable | rho | p | pBH | rho | p | pBH |
| O_CpG_1.2 | socm_sum | 0.19 | 0.116 | 0.538 | 0.07 | 0.572 | 0.817 |
| O_CpG_1.2 | FERUS_SUM | 0.08 | 0.471 | 0.974 | -0.02 | 0.866 | 0.964 |
| O_CpG_1.2 | siat_total | -0.10 | 0.392 | 0.960 | 0.11 | 0.329 | 0.706 |
| O_CpG_1.2 | siat_loc | -0.11 | 0.341 | 0.888 | 0.11 | 0.341 | 0.706 |
| O_CpG_1.2 | siat_craving | -0.05 | 0.678 | 0.976 | 0.10 | 0.403 | 0.733 |
| O_CpG_1.2 | mrnaOXT | -0.03 | 0.828 | 0.998 | -0.05 | 0.734 | 0.947 |
| O_CpG_13 | socm_sum | 0.10 | 0.421 | 0.974 | 0.04 | 0.765 | 0.955 |
| O_CpG_13 | FERUS_SUM | 0.13 | 0.275 | 0.888 | -0.02 | 0.846 | 0.964 |
| O_CpG_13 | siat_total | 0.00 | 0.998 | 0.998 | 0.07 | 0.559 | 0.817 |
| O_CpG_13 | siat_loc | -0.01 | 0.956 | 0.998 | 0.07 | 0.579 | 0.818 |
| O_CpG_13 | siat_craving | 0.05 | 0.665 | 0.976 | 0.04 | 0.753 | 0.955 |
| O_CpG_13 | mrnaOXT | -0.05 | 0.673 | 0.976 | -0.10 | 0.510 | 0.795 |
| O_CpG_14.15 | socm_sum | 0.04 | 0.743 | 0.984 | 0.09 | 0.480 | 0.778 |
| O_CpG_14.15 | FERUS_SUM | 0.08 | 0.506 | 0.976 | -0.06 | 0.594 | 0.827 |
| O_CpG_14.15 | siat_total | -0.05 | 0.695 | 0.976 | 0.15 | 0.185 | 0.567 |
| O_CpG_14.15 | siat_loc | -0.06 | 0.629 | 0.976 | 0.12 | 0.309 | 0.706 |
| O_CpG_14.15 | siat_craving | 0.02 | 0.883 | 0.998 | 0.19 | 0.102 | 0.422 |
| O_CpG_14.15 | mrnaOXT | 0.04 | 0.747 | 0.984 | 0.11 | 0.431 | 0.739 |
| O_CpG_16 | socm_sum | 0.19 | 0.103 | 0.517 | 0.12 | 0.327 | 0.706 |
| O_CpG_16 | FERUS_SUM | 0.04 | 0.708 | 0.976 | -0.16 | 0.160 | 0.548 |
| O_CpG_16 | siat_total | -0.06 | 0.615 | 0.976 | 0.10 | 0.388 | 0.727 |
| O_CpG_16 | siat_loc | -0.06 | 0.612 | 0.976 | 0.09 | 0.467 | 0.768 |
| O_CpG_16 | siat_craving | -0.04 | 0.719 | 0.981 | 0.14 | 0.215 | 0.599 |
| O_CpG_16 | mrnaOXT | 0.01 | 0.956 | 0.998 | 0.21 | 0.136 | 0.479 |
| O_CpG_18 | socm_sum | 0.21 | 0.087 | 0.455 | -0.16 | 0.232 | 0.632 |
| O_CpG_18 | FERUS_SUM | 0.05 | 0.671 | 0.976 | -0.11 | 0.383 | 0.727 |
| O_CpG_18 | siat_total | 0.15 | 0.234 | 0.867 | -0.01 | 0.955 | 0.984 |
| O_CpG_18 | siat_loc | 0.13 | 0.305 | 0.888 | -0.02 | 0.853 | 0.964 |
| O_CpG_18 | siat_craving | 0.16 | 0.190 | 0.762 | 0.00 | 0.971 | 0.988 |
| O_CpG_18 | mrnaOXT | -0.06 | 0.626 | 0.976 | -0.15 | 0.347 | 0.706 |
| O_CpG_19 | socm_sum | 0.06 | 0.641 | 0.976 | 0.12 | 0.375 | 0.725 |
| O_CpG_19 | FERUS_SUM | 0.09 | 0.470 | 0.974 | 0.01 | 0.931 | 0.984 |
| O_CpG_19 | siat_total | -0.02 | 0.870 | 0.998 | 0.01 | 0.960 | 0.984 |
| O_CpG_19 | siat_loc | -0.03 | 0.829 | 0.998 | 0.01 | 0.958 | 0.984 |
| O_CpG_19 | siat_craving | 0.01 | 0.955 | 0.998 | 0.02 | 0.869 | 0.964 |
| O_CpG_19 | mrnaOXT | -0.04 | 0.741 | 0.984 | 0.03 | 0.850 | 0.964 |
| O_CpG_24.25 | socm_sum | 0.00 | 0.984 | 0.998 | 0.10 | 0.445 | 0.742 |
| O_CpG_24.25 | FERUS_SUM | 0.00 | 0.995 | 0.998 | -0.10 | 0.414 | 0.739 |
| O_CpG_24.25 | siat_total | -0.13 | 0.293 | 0.888 | 0.04 | 0.773 | 0.955 |
| O_CpG_24.25 | siat_loc | -0.12 | 0.327 | 0.888 | 0.03 | 0.844 | 0.964 |
| O_CpG_24.25 | siat_craving | -0.12 | 0.340 | 0.888 | 0.05 | 0.687 | 0.906 |
| O_CpG_24.25 | mrnaOXT | 0.01 | 0.950 | 0.998 | 0.00 | 0.996 | 0.996 |
| O_CpG_26 | socm_sum | -0.02 | 0.871 | 0.998 | 0.02 | 0.876 | 0.964 |
| O_CpG_26 | FERUS_SUM | 0.08 | 0.460 | 0.974 | -0.11 | 0.355 | 0.706 |
| O_CpG_26 | siat_total | -0.08 | 0.467 | 0.974 | 0.02 | 0.891 | 0.972 |
| O_CpG_26 | siat_loc | -0.06 | 0.573 | 0.976 | -0.04 | 0.730 | 0.947 |
| O_CpG_26 | siat_craving | -0.13 | 0.260 | 0.867 | 0.11 | 0.342 | 0.706 |
| O_CpG_26 | mrnaOXT | -0.09 | 0.465 | 0.974 | 0.01 | 0.936 | 0.984 |
| O_CpG_3 | socm_sum | 0.24 | 0.041 | 0.245 | 0.09 | 0.445 | 0.742 |
| O_CpG_3 | FERUS_SUM | 0.30 | 0.008 | 0.048 | -0.08 | 0.493 | 0.789 |
| O_CpG_3 | siat_total | 0.02 | 0.880 | 0.998 | 0.14 | 0.245 | 0.632 |
| O_CpG_3 | siat_loc | 0.00 | 0.975 | 0.998 | 0.15 | 0.192 | 0.567 |
| O_CpG_3 | siat_craving | 0.03 | 0.802 | 0.998 | 0.08 | 0.517 | 0.796 |
| O_CpG_3 | mrnaOXT | -0.04 | 0.758 | 0.988 | -0.07 | 0.622 | 0.844 |
| O_CpG_4 | socm_sum | 0.14 | 0.233 | 0.867 | 0.17 | 0.173 | 0.567 |
| O_CpG_4 | FERUS_SUM | 0.16 | 0.173 | 0.717 | -0.10 | 0.397 | 0.732 |
| O_CpG_4 | siat_total | -0.05 | 0.673 | 0.976 | 0.23 | 0.046 | 0.219 |
| O_CpG_4 | siat_loc | -0.06 | 0.609 | 0.976 | 0.23 | 0.055 | 0.252 |
| O_CpG_4 | siat_craving | 0.01 | 0.928 | 0.998 | 0.19 | 0.114 | 0.456 |
| O_CpG_4 | mrnaOXT | -0.24 | 0.051 | 0.290 | 0.00 | 0.994 | 0.996 |
| O_CpG_5 | socm_sum | 0.14 | 0.257 | 0.867 | 0.07 | 0.599 | 0.827 |
| O_CpG_5 | FERUS_SUM | 0.18 | 0.139 | 0.618 | 0.10 | 0.431 | 0.739 |
| O_CpG_5 | siat_total | -0.10 | 0.426 | 0.974 | 0.17 | 0.185 | 0.567 |
| O_CpG_5 | siat_loc | -0.11 | 0.386 | 0.960 | 0.13 | 0.290 | 0.706 |
| O_CpG_5 | siat_craving | 0.01 | 0.945 | 0.998 | 0.19 | 0.130 | 0.473 |
| O_CpG_5 | mrnaOXT | -0.13 | 0.329 | 0.888 | 0.10 | 0.502 | 0.792 |
| O_CpG_7.8 | socm_sum | 0.17 | 0.157 | 0.673 | 0.02 | 0.876 | 0.964 |
| O_CpG_7.8 | FERUS_SUM | 0.13 | 0.251 | 0.867 | 0.01 | 0.917 | 0.983 |
| O_CpG_7.8 | siat_total | -0.11 | 0.337 | 0.888 | 0.12 | 0.303 | 0.706 |
| O_CpG_7.8 | siat_loc | -0.13 | 0.259 | 0.867 | 0.15 | 0.194 | 0.567 |
| O_CpG_7.8 | siat_craving | -0.01 | 0.924 | 0.998 | 0.06 | 0.626 | 0.844 |
| O_CpG_7.8 | mrnaOXT | -0.06 | 0.619 | 0.976 | 0.11 | 0.430 | 0.739 |
| O_CpG_9.10 | socm_sum | 0.19 | 0.117 | 0.538 | 0.08 | 0.530 | 0.804 |
| O_CpG_9.10 | FERUS_SUM | 0.20 | 0.076 | 0.412 | -0.07 | 0.569 | 0.817 |
| O_CpG_9.10 | siat_total | -0.05 | 0.689 | 0.976 | 0.12 | 0.296 | 0.706 |
| O_CpG_9.10 | siat_loc | -0.06 | 0.621 | 0.976 | 0.11 | 0.359 | 0.706 |
| O_CpG_9.10 | siat_craving | 0.01 | 0.961 | 0.998 | 0.12 | 0.326 | 0.706 |
| O_CpG_9.10 | mrnaOXT | -0.13 | 0.303 | 0.888 | 0.04 | 0.780 | 0.955 |
| mrnaOXT | socm_sum | -0.07 | 0.610 | 0.976 | 0.17 | 0.247 | 0.632 |
| mrnaOXT | FERUS_SUM | -0.08 | 0.511 | 0.976 | 0.03 | 0.821 | 0.964 |
| mrnaOXT | siat_total | 0.00 | 0.975 | 0.998 | 0.13 | 0.356 | 0.706 |
| mrnaOXT | siat_loc | -0.05 | 0.706 | 0.976 | 0.08 | 0.572 | 0.817 |
| mrnaOXT | siat_craving | 0.05 | 0.703 | 0.976 | 0.18 | 0.199 | 0.567 |

**Table S4.** Spearman correlation coefficients between methylation and IUD tendencies, social support and mRNA abundance for SLC6A4

|  |  | HC | | | MDD | | |
| --- | --- | --- | --- | --- | --- | --- | --- |
| CpG-unit | Variable | rho | p | pBH | rho | p | pBH |
| S_CpG_1 | FERUS_SUM | -0,08 | 0,468 | 0,922 | -0,04 | 0,708 | 0,960 |
| S_CpG_1 | mrnaSLC | 0,05 | 0,709 | 0,841 | -0,08 | 0,584 | 0,771 |
| S_CpG_1 | siat_craving | 0,02 | 0,890 | 0,890 | -0,15 | 0,207 | 0,622 |
| S_CpG_1 | siat_loc | 0,05 | 0,643 | 0,885 | -0,04 | 0,745 | 0,850 |
| S_CpG_1 | siat_total | 0,05 | 0,634 | 0,812 | -0,08 | 0,498 | 0,856 |
| S_CpG_1 | socm_sum | -0,03 | 0,782 | 0,829 | -0,19 | 0,107 | 0,238 |
| S_CpG_10 | FERUS_SUM | -0,05 | 0,659 | 0,922 | -0,09 | 0,444 | 0,917 |
| S_CpG_10 | mrnaSLC | 0,08 | 0,521 | 0,717 | 0,20 | 0,152 | 0,385 |
| S_CpG_10 | siat_craving | 0,08 | 0,505 | 0,666 | 0,09 | 0,431 | 0,678 |
| S_CpG_10 | siat_loc | 0,14 | 0,236 | 0,675 | 0,01 | 0,926 | 0,926 |
| S_CpG_10 | siat_total | 0,13 | 0,258 | 0,616 | 0,05 | 0,646 | 0,889 |
| S_CpG_10 | socm_sum | 0,17 | 0,164 | 0,338 | -0,02 | 0,899 | 0,957 |
| S_CpG_11-15 | FERUS_SUM | 0,01 | 0,931 | 0,983 | 0,01 | 0,898 | 0,960 |
| S_CpG_11-15 | mrnaSLC | -0,15 | 0,240 | 0,705 | 0,29 | 0,035 | 0,185 |
| S_CpG_11-15 | siat_craving | -0,11 | 0,321 | 0,623 | -0,20 | 0,090 | 0,493 |
| S_CpG_11-15 | siat_loc | -0,25 | 0,027 | 0,140 | -0,16 | 0,166 | 0,547 |
| S_CpG_11-15 | siat_total | -0,23 | 0,041 | 0,191 | -0,17 | 0,136 | 0,450 |
| S_CpG_11-15 | socm_sum | 0,03 | 0,803 | 0,829 | -0,16 | 0,192 | 0,317 |
| S_CpG_16.17 | FERUS_SUM | -0,11 | 0,340 | 0,883 | 0,01 | 0,904 | 0,960 |
| S_CpG_16.17 | mrnaSLC | -0,01 | 0,928 | 0,928 | 0,07 | 0,621 | 0,771 |
| S_CpG_16.17 | siat_craving | 0,06 | 0,628 | 0,752 | -0,17 | 0,147 | 0,592 |
| S_CpG_16.17 | siat_loc | 0,02 | 0,845 | 0,931 | -0,19 | 0,100 | 0,378 |
| S_CpG_16.17 | siat_total | 0,03 | 0,770 | 0,873 | -0,19 | 0,102 | 0,413 |
| S_CpG_16.17 | socm_sum | 0,08 | 0,503 | 0,639 | -0,24 | 0,050 | 0,229 |
| S_CpG_18.19 | FERUS_SUM | -0,01 | 0,901 | 0,983 | -0,05 | 0,647 | 0,960 |
| S_CpG_18.19 | mrnaSLC | -0,12 | 0,337 | 0,705 | 0,32 | 0,018 | 0,148 |
| S_CpG_18.19 | siat_craving | 0,09 | 0,435 | 0,666 | -0,16 | 0,162 | 0,592 |
| S_CpG_18.19 | siat_loc | -0,02 | 0,831 | 0,931 | -0,19 | 0,103 | 0,378 |
| S_CpG_18.19 | siat_total | 0,02 | 0,833 | 0,887 | -0,18 | 0,113 | 0,413 |
| S_CpG_18.19 | socm_sum | 0,11 | 0,345 | 0,526 | -0,20 | 0,102 | 0,238 |
| S_CpG_28 | FERUS_SUM | -0,14 | 0,220 | 0,883 | -0,13 | 0,252 | 0,831 |
| S_CpG_28 | mrnaSLC | -0,10 | 0,411 | 0,705 | 0,09 | 0,518 | 0,744 |
| S_CpG_28 | siat_craving | -0,09 | 0,441 | 0,666 | 0,02 | 0,890 | 0,998 |
| S_CpG_28 | siat_loc | -0,02 | 0,851 | 0,931 | -0,08 | 0,491 | 0,792 |
| S_CpG_28 | siat_total | -0,05 | 0,640 | 0,812 | -0,05 | 0,674 | 0,889 |
| S_CpG_28 | socm_sum | 0,13 | 0,269 | 0,467 | -0,04 | 0,753 | 0,892 |
| S_CpG_29.30 | FERUS_SUM | -0,16 | 0,172 | 0,883 | 0,03 | 0,802 | 0,960 |
| S_CpG_29.30 | mrnaSLC | 0,16 | 0,202 | 0,705 | 0,27 | 0,049 | 0,185 |
| S_CpG_29.30 | siat_craving | 0,08 | 0,503 | 0,666 | 0,01 | 0,944 | 0,998 |
| S_CpG_29.30 | siat_loc | 0,04 | 0,742 | 0,931 | 0,08 | 0,482 | 0,792 |
| S_CpG_29.30 | siat_total | 0,04 | 0,704 | 0,860 | 0,05 | 0,665 | 0,889 |
| S_CpG_29.30 | socm_sum | 0,17 | 0,155 | 0,338 | -0,10 | 0,410 | 0,572 |
| S_CpG_3 | FERUS_SUM | 0,11 | 0,347 | 0,883 | -0,03 | 0,791 | 0,960 |
| S_CpG_3 | mrnaSLC | -0,11 | 0,365 | 0,705 | 0,46 | 0,001 | 0,006 |
| S_CpG_3 | siat_craving | 0,05 | 0,638 | 0,752 | -0,02 | 0,889 | 0,998 |
| S_CpG_3 | siat_loc | -0,03 | 0,779 | 0,931 | 0,06 | 0,611 | 0,792 |
| S_CpG_3 | siat_total | -0,01 | 0,965 | 0,965 | 0,01 | 0,916 | 0,970 |
| S_CpG_3 | socm_sum | 0,06 | 0,588 | 0,718 | -0,13 | 0,274 | 0,430 |
| S_CpG_31.32 | FERUS_SUM | 0,04 | 0,719 | 0,922 | -0,03 | 0,820 | 0,960 |
| S_CpG_31.32 | mrnaSLC | -0,13 | 0,345 | 0,705 | -0,18 | 0,229 | 0,539 |
| S_CpG_31.32 | siat_craving | -0,25 | 0,036 | 0,171 | -0,10 | 0,421 | 0,678 |
| S_CpG_31.32 | siat_loc | -0,27 | 0,023 | 0,140 | -0,15 | 0,214 | 0,590 |
| S_CpG_31.32 | siat_total | -0,27 | 0,022 | 0,147 | -0,12 | 0,320 | 0,821 |
| S_CpG_31.32 | socm_sum | -0,16 | 0,208 | 0,381 | -0,07 | 0,568 | 0,721 |
| S_CpG_39-42 | FERUS_SUM | 0,15 | 0,229 | 0,883 | -0,18 | 0,142 | 0,745 |
| S_CpG_39-42 | mrnaSLC | -0,11 | 0,391 | 0,705 | 0,01 | 0,939 | 0,939 |
| S_CpG_39-42 | siat_craving | -0,09 | 0,463 | 0,666 | -0,12 | 0,344 | 0,651 |
| S_CpG_39-42 | siat_loc | -0,16 | 0,200 | 0,675 | -0,11 | 0,393 | 0,792 |
| S_CpG_39-42 | siat_total | -0,12 | 0,306 | 0,616 | -0,12 | 0,342 | 0,821 |
| S_CpG_39-42 | socm_sum | -0,27 | 0,030 | 0,123 | -0,22 | 0,076 | 0,238 |
| S_CpG_4 | FERUS_SUM | 0,17 | 0,151 | 0,883 | 0,05 | 0,662 | 0,960 |
| S_CpG_4 | mrnaSLC | -0,11 | 0,390 | 0,705 | 0,05 | 0,711 | 0,782 |
| S_CpG_4 | siat_craving | -0,19 | 0,105 | 0,348 | 0,00 | 0,986 | 0,998 |
| S_CpG_4 | siat_loc | -0,09 | 0,440 | 0,787 | -0,07 | 0,566 | 0,792 |
| S_CpG_4 | siat_total | -0,13 | 0,273 | 0,616 | -0,05 | 0,649 | 0,889 |
| S_CpG_4 | socm_sum | -0,11 | 0,371 | 0,526 | -0,09 | 0,461 | 0,608 |
| S_CpG_45 | FERUS_SUM | 0,13 | 0,275 | 0,883 | -0,12 | 0,328 | 0,835 |
| S_CpG_45 | mrnaSLC | 0,12 | 0,373 | 0,705 | 0,04 | 0,763 | 0,812 |
| S_CpG_45 | siat_craving | -0,14 | 0,236 | 0,537 | -0,12 | 0,324 | 0,651 |
| S_CpG_45 | siat_loc | 0,01 | 0,915 | 0,943 | -0,16 | 0,203 | 0,590 |
| S_CpG_45 | siat_total | -0,04 | 0,767 | 0,873 | -0,16 | 0,196 | 0,588 |
| S_CpG_45 | socm_sum | -0,24 | 0,052 | 0,172 | -0,19 | 0,130 | 0,238 |
| S_CpG_46 | FERUS_SUM | 0,18 | 0,130 | 0,883 | 0,17 | 0,161 | 0,745 |
| S_CpG_46 | mrnaSLC | 0,09 | 0,476 | 0,705 | -0,27 | 0,063 | 0,206 |
| S_CpG_46 | siat_craving | -0,02 | 0,842 | 0,890 | -0,02 | 0,860 | 0,998 |
| S_CpG_46 | siat_loc | -0,02 | 0,875 | 0,931 | 0,07 | 0,600 | 0,792 |
| S_CpG_46 | siat_total | -0,03 | 0,793 | 0,873 | 0,04 | 0,748 | 0,901 |
| S_CpG_46 | socm_sum | -0,11 | 0,358 | 0,526 | 0,24 | 0,055 | 0,229 |
| S_CpG_47.48 | FERUS_SUM | 0,08 | 0,524 | 0,922 | -0,11 | 0,347 | 0,835 |
| S_CpG_47.48 | mrnaSLC | 0,02 | 0,870 | 0,897 | 0,15 | 0,301 | 0,622 |
| S_CpG_47.48 | siat_craving | -0,22 | 0,068 | 0,251 | -0,09 | 0,474 | 0,680 |
| S_CpG_47.48 | siat_loc | -0,05 | 0,687 | 0,907 | -0,07 | 0,575 | 0,792 |
| S_CpG_47.48 | siat_total | -0,09 | 0,442 | 0,729 | -0,09 | 0,442 | 0,856 |
| S_CpG_47.48 | socm_sum | -0,19 | 0,122 | 0,310 | -0,20 | 0,117 | 0,238 |
| S_CpG_5.6 | FERUS_SUM | -0,06 | 0,615 | 0,922 | 0,00 | 0,994 | 0,994 |
| S_CpG_5.6 | mrnaSLC | -0,18 | 0,145 | 0,705 | 0,47 | 0,001 | 0,006 |
| S_CpG_5.6 | siat_craving | 0,06 | 0,632 | 0,752 | 0,00 | 0,968 | 0,998 |
| S_CpG_5.6 | siat_loc | 0,13 | 0,245 | 0,675 | 0,02 | 0,837 | 0,891 |
| S_CpG_5.6 | siat_total | 0,12 | 0,296 | 0,616 | 0,00 | 0,969 | 0,970 |
| S_CpG_5.6 | socm_sum | 0,17 | 0,159 | 0,338 | -0,04 | 0,769 | 0,892 |
| S_CpG_52 | FERUS_SUM | 0,17 | 0,171 | 0,883 | -0,16 | 0,181 | 0,745 |
| S_CpG_52 | mrnaSLC | 0,03 | 0,832 | 0,897 | -0,28 | 0,051 | 0,185 |
| S_CpG_52 | siat_craving | -0,14 | 0,244 | 0,537 | 0,12 | 0,346 | 0,651 |
| S_CpG_52 | siat_loc | -0,07 | 0,570 | 0,818 | -0,09 | 0,473 | 0,792 |
| S_CpG_52 | siat_total | -0,09 | 0,480 | 0,754 | 0,00 | 0,970 | 0,970 |
| S_CpG_52 | socm_sum | 0,04 | 0,718 | 0,817 | 0,04 | 0,784 | 0,892 |
| S_CpG_53.54 | FERUS_SUM | 0,16 | 0,182 | 0,883 | -0,15 | 0,246 | 0,831 |
| S_CpG_53.54 | mrnaSLC | 0,09 | 0,491 | 0,705 | -0,07 | 0,654 | 0,771 |
| S_CpG_53.54 | siat_craving | -0,16 | 0,181 | 0,497 | -0,12 | 0,375 | 0,651 |
| S_CpG_53.54 | siat_loc | -0,15 | 0,217 | 0,675 | -0,10 | 0,439 | 0,792 |
| S_CpG_53.54 | siat_total | -0,16 | 0,193 | 0,616 | -0,12 | 0,373 | 0,821 |
| S_CpG_53.54 | socm_sum | -0,26 | 0,039 | 0,143 | -0,32 | 0,018 | 0,116 |
| S_CpG_57.58 | FERUS_SUM | 0,06 | 0,641 | 0,922 | -0,11 | 0,387 | 0,850 |
| S_CpG_57.58 | mrnaSLC | -0,15 | 0,252 | 0,705 | -0,10 | 0,518 | 0,744 |
| S_CpG_57.58 | siat_craving | -0,13 | 0,283 | 0,584 | -0,16 | 0,188 | 0,622 |
| S_CpG_57.58 | siat_loc | -0,26 | 0,030 | 0,140 | -0,20 | 0,101 | 0,378 |
| S_CpG_57.58 | siat_total | -0,24 | 0,048 | 0,198 | -0,22 | 0,076 | 0,357 |
| S_CpG_57.58 | socm_sum | -0,32 | 0,009 | 0,057 | -0,01 | 0,957 | 0,983 |
| S_CpG_60 | FERUS_SUM | 0,00 | 0,991 | 0,991 | 0,01 | 0,931 | 0,960 |
| S_CpG_60 | mrnaSLC | -0,02 | 0,865 | 0,897 | -0,21 | 0,145 | 0,385 |
| S_CpG_60 | siat_craving | 0,04 | 0,732 | 0,833 | 0,12 | 0,349 | 0,651 |
| S_CpG_60 | siat_loc | -0,08 | 0,525 | 0,787 | -0,03 | 0,785 | 0,863 |
| S_CpG_60 | siat_total | -0,02 | 0,865 | 0,892 | 0,04 | 0,753 | 0,901 |
| S_CpG_60 | socm_sum | 0,01 | 0,923 | 0,923 | 0,02 | 0,849 | 0,934 |
| S_CpG_61-63 | FERUS_SUM | 0,04 | 0,727 | 0,922 | -0,03 | 0,828 | 0,960 |
| S_CpG_61-63 | mrnaSLC | 0,03 | 0,821 | 0,897 | -0,03 | 0,848 | 0,875 |
| S_CpG_61-63 | siat_craving | -0,26 | 0,029 | 0,171 | 0,01 | 0,965 | 0,998 |
| S_CpG_61-63 | siat_loc | -0,13 | 0,285 | 0,723 | -0,12 | 0,345 | 0,792 |
| S_CpG_61-63 | siat_total | -0,17 | 0,161 | 0,592 | -0,07 | 0,560 | 0,881 |
| S_CpG_61-63 | socm_sum | -0,22 | 0,080 | 0,239 | -0,13 | 0,320 | 0,481 |
| S_CpG_64 | FERUS_SUM | 0,01 | 0,953 | 0,983 | -0,04 | 0,754 | 0,960 |
| S_CpG_64 | mrnaSLC | -0,06 | 0,641 | 0,841 | -0,17 | 0,252 | 0,554 |
| S_CpG_64 | siat_craving | -0,15 | 0,210 | 0,533 | -0,11 | 0,355 | 0,651 |
| S_CpG_64 | siat_loc | 0,00 | 0,971 | 0,971 | -0,28 | 0,021 | 0,113 |
| S_CpG_64 | siat_total | -0,06 | 0,624 | 0,812 | -0,22 | 0,075 | 0,357 |
| S_CpG_64 | socm_sum | -0,20 | 0,112 | 0,307 | -0,26 | 0,035 | 0,195 |
| S_CpG_65 | FERUS_SUM | -0,07 | 0,578 | 0,922 | 0,12 | 0,354 | 0,835 |
| S_CpG_65 | mrnaSLC | -0,15 | 0,250 | 0,705 | -0,33 | 0,026 | 0,173 |
| S_CpG_65 | siat_craving | -0,24 | 0,054 | 0,224 | -0,12 | 0,362 | 0,651 |
| S_CpG_65 | siat_loc | -0,08 | 0,521 | 0,787 | -0,14 | 0,290 | 0,736 |
| S_CpG_65 | siat_total | -0,15 | 0,224 | 0,616 | -0,11 | 0,406 | 0,838 |
| S_CpG_65 | socm_sum | -0,17 | 0,192 | 0,372 | -0,11 | 0,416 | 0,572 |
| S_CpG_66 | FERUS_SUM | 0,06 | 0,650 | 0,922 | -0,02 | 0,876 | 0,960 |
| S_CpG_66 | mrnaSLC | -0,10 | 0,441 | 0,705 | -0,13 | 0,390 | 0,715 |
| S_CpG_66 | siat_craving | -0,10 | 0,421 | 0,666 | -0,18 | 0,150 | 0,592 |
| S_CpG_66 | siat_loc | -0,11 | 0,354 | 0,787 | -0,08 | 0,515 | 0,792 |
| S_CpG_66 | siat_total | -0,12 | 0,317 | 0,616 | -0,11 | 0,363 | 0,821 |
| S_CpG_66 | socm_sum | -0,29 | 0,018 | 0,085 | -0,20 | 0,111 | 0,238 |
| S_CpG_69 | FERUS_SUM | -0,13 | 0,302 | 0,883 | -0,06 | 0,601 | 0,960 |
| S_CpG_69 | mrnaSLC | 0,09 | 0,490 | 0,705 | -0,14 | 0,327 | 0,635 |
| S_CpG_69 | siat_craving | 0,08 | 0,499 | 0,666 | -0,12 | 0,341 | 0,651 |
| S_CpG_69 | siat_loc | 0,11 | 0,367 | 0,787 | -0,06 | 0,647 | 0,792 |
| S_CpG_69 | siat_total | 0,09 | 0,436 | 0,729 | -0,08 | 0,514 | 0,856 |
| S_CpG_69 | socm_sum | -0,03 | 0,781 | 0,829 | -0,18 | 0,147 | 0,255 |
| S_CpG_7 | FERUS_SUM | -0,03 | 0,844 | 0,981 | -0,15 | 0,277 | 0,832 |
| S_CpG_7 | mrnaSLC | 0,12 | 0,389 | 0,705 | -0,12 | 0,474 | 0,744 |
| S_CpG_7 | siat_craving | 0,02 | 0,872 | 0,890 | -0,08 | 0,570 | 0,784 |
| S_CpG_7 | siat_loc | -0,09 | 0,462 | 0,787 | 0,07 | 0,601 | 0,792 |
| S_CpG_7 | siat_total | -0,07 | 0,598 | 0,812 | 0,02 | 0,886 | 0,970 |
| S_CpG_7 | socm_sum | -0,11 | 0,383 | 0,526 | -0,23 | 0,089 | 0,238 |
| S_CpG_70.71 | FERUS_SUM | 0,04 | 0,722 | 0,922 | -0,07 | 0,572 | 0,960 |
| S_CpG_70.71 | mrnaSLC | 0,05 | 0,709 | 0,841 | -0,29 | 0,046 | 0,185 |
| S_CpG_70.71 | siat_craving | -0,25 | 0,033 | 0,171 | 0,00 | 0,971 | 0,998 |
| S_CpG_70.71 | siat_loc | -0,23 | 0,055 | 0,226 | 0,04 | 0,747 | 0,850 |
| S_CpG_70.71 | siat_total | -0,25 | 0,038 | 0,191 | 0,04 | 0,764 | 0,901 |
| S_CpG_70.71 | socm_sum | -0,29 | 0,018 | 0,085 | -0,20 | 0,127 | 0,238 |
| S_CpG_8.9 | FERUS_SUM | -0,02 | 0,863 | 0,981 | -0,08 | 0,504 | 0,960 |
| S_CpG_8.9 | mrnaSLC | 0,16 | 0,188 | 0,705 | -0,05 | 0,701 | 0,782 |
| S_CpG_8.9 | siat_craving | 0,10 | 0,380 | 0,666 | 0,00 | 0,998 | 0,998 |
| S_CpG_8.9 | siat_loc | 0,08 | 0,491 | 0,787 | 0,02 | 0,893 | 0,921 |
| S_CpG_8.9 | siat_total | 0,11 | 0,353 | 0,647 | -0,01 | 0,963 | 0,970 |
| S_CpG_8.9 | socm_sum | 0,09 | 0,439 | 0,580 | 0,00 | 0,983 | 0,983 |
| mrnaSLC | FERUS_SUM | -0,09 | 0,452 | 0,922 | 0,07 | 0,596 | 0,960 |
| mrnaSLC | siat_craving | 0,19 | 0,120 | 0,360 | -0,10 | 0,472 | 0,680 |
| mrnaSLC | siat_loc | 0,09 | 0,460 | 0,787 | -0,06 | 0,648 | 0,792 |
| mrnaSLC | siat_total | 0,13 | 0,290 | 0,616 | -0,09 | 0,519 | 0,856 |
| mrnaSLC | socm_sum | 0,05 | 0,714 | 0,817 | -0,22 | 0,126 | 0,238 |

**Linear mixed effects models**

*FERUS*

DV: OXT methylation

**Table S5.** Estimates and CI values for associations with OXT methylation difference (t1-t0).

| *Predictors* | *β* | *CI* | *p* |
| --- | --- | --- | --- |
| (Intercept) | 0.0083 | -0.0527 – 0.0698 | 0.644 |
| NG | 0.0093 | -0.0247 – 0.0440 | 0.287 |
| Lymph | -0.0134 | -0.0464 – 0.0190 | 0.123 |
| BDI-II | -0.0103 | -0.0676 – 0.0418 | 0.482 |
| AD | -0.0073 | -0.0475 – 0.0290 | 0.494 |
| BMI | -0.0021 | -0.0340 – 0.0281 | 0.811 |
| Nicotine | 0.0029 | -0.0306 – 0.0366 | 0.779 |
| FERUS | 0.0202 | -0.0456 – 0.0753 | 0.160 |
| Group | -0.0047 | -0.1085 – 0.0991 | 0.879 |
| FERUS × Group | -0.0328 | -0.1067 – 0.0407 | 0.089 |

Note. BMI: Body Mass Index; BDI-II: baseline depression severity; AD: baseline dose equivalents of antidepressants; NG: neutrophilic granulocytes; Lymph: lymphocytes; FERUS: Social network sum score; reference level for group was MDD.

DV: SLC6A4 methylation

**Table S6.** Estimates, CIs and p-values for associations with SLC6A4 methylation difference (t1-t0).

| *Predictors* | *β* | *CI* | *p* |
| --- | --- | --- | --- |
| (Intercept) | 0.0110 | -0.0113 – 0.0345 | 0.084 |
| NG | -0.0009 | -0.0124 – 0.0106 | 0.767 |
| Lymph | -0.0012 | -0.0101 – 0.0098 | 0.680 |
| BDI-II | -0.0029 | -0.0201 – 0.0145 | 0.525 |
| AD | -0.0013 | -0.0136 – 0.0117 | 0.693 |
| BMI | 0.0004 | -0.0121 – 0.0124 | 0.909 |
| Nicotine | 0.0007 | -0.0118 – 0.0111 | 0.828 |
| FERUS | -0.0072 | -0.0236 – 0.0119 | 0.151 |
| Group | -0.0030 | -0.0374 – 0.0321 | 0.767 |
| FERUS × Group | 0.0059 | -0.0171 – 0.0262 | 0.306 |

Note. BMI: Body Mass Index; BDI-II: baseline depression severity; AD: baseline dose equivalents of antidepressants; NG: neutrophilic granulocytes; Lymph: lymphocytes; FERUS: Social network sum score; reference level for group was MDD.

DV: NR3C1 methylation

**Table S7.** Estimates, CIs and p-values for associations with NR3C1 methylation difference (t1-t0).

| *Predictors* | *β* | *CI* | *p* |
| --- | --- | --- | --- |
| (Intercept) | -0.0057 | -0.0244 – 0.0143 | 0.313 |
| NG | -0.0003 | -0.0070 – 0.0067 | 0.896 |
| Lymph | -0.0006 | -0.0097 – 0.0072 | 0.817 |
| BDI-II | -0.0044 | -0.0168 – 0.0081 | 0.190 |
| AD | 0.0020 | -0.0063 – 0.0111 | 0.444 |
| BMI | -0.0035 | -0.0123 – 0.0041 | 0.128 |
| Nicotine | 0.0046 | -0.0059 – 0.0135 | 0.041 |
| FERUS | 0.0011 | -0.0124 – 0.0145 | 0.743 |
| Group | 0.0104 | -0.0206 – 0.0387 | 0.172 |
| FERUS × Group | 0.0004 | -0.0168 – 0.0160 | 0.922 |

Note. BMI: Body Mass Index; BDI-II: baseline depression severity; AD: baseline dose equivalents of antidepressants; NG: neutrophilic granulocytes; Lymph: lymphocytes; FERUS: Social network sum score; reference level for group was MDD.

S-IAT total score

DV: OXT methylation

**Table S8.** Estimates, CIs and p-values for associations with OXT methylation difference (t1-t0).

| *Predictors* | *β* | *CI* | *p* |
| --- | --- | --- | --- |
| (Intercept) | 0.0131 | -0.0492 – 0.0776 | 0.453 |
| NG | 0.0146 | -0.0195 – 0.0491 | 0.103 |
| Lymph | -0.0144 | -0.0424 – 0.0173 | 0.108 |
| BDI-II | -0.0112 | -0.0723 – 0.0402 | 0.430 |
| AD | -0.0091 | -0.0494 – 0.0419 | 0.402 |
| BMI | -0.0073 | -0.0438 – 0.0298 | 0.419 |
| Nicotine | 0.0040 | -0.0331 – 0.0370 | 0.678 |
| s-IAT | -0.0156 | -0.0506 – 0.0210 | 0.119 |
| Group | -0.0017 | -0.1221 – 0.1186 | 0.954 |
| s-IAT × Group | 0.0138 | -0.0409 – 0.0651 | 0.361 |

Note. BMI: Body Mass Index; BDI-II: baseline depression severity; AD: baseline dose equivalents of antidepressants; NG: neutrophilic granulocytes; Lymph: lymphocytes; s-IAT: internet addiction total score; reference level for group was MDD.

DV: SLC6A4 methylation

**Table S9.** Estimates, CIs and p-values for associations with SLC6A4 methylation difference (t1-t0).

| *Predictors* | *β* | *CI* | *p* |
| --- | --- | --- | --- |
| (Intercept) | 0.0108 | -0.0163 – 0.0332 | 0.105 |
| NG | -0.0014 | -0.0120 – 0.0102 | 0.651 |
| Lymph | -0.0008 | -0.0113 – 0.0103 | 0.778 |
| BDI-II | 0.0004 | -0.0181 – 0.0205 | 0.938 |
| AD | -0.0013 | -0.0133 – 0.0139 | 0.720 |
| BMI | 0.0009 | -0.0106 – 0.0114 | 0.777 |
| Nicotine | 0.0009 | -0.0116 – 0.0114 | 0.763 |
| s-IAT | 0.0002 | -0.0121 – 0.0133 | 0.952 |
| Group | -0.0050 | -0.0494 – 0.0367 | 0.634 |
| s-IAT × Group | -0.0020 | -0.0221 – 0.0172 | 0.704 |

Note. BMI: Body Mass Index; BDI-II: baseline depression severity; AD: baseline dose equivalents of antidepressants; NG: neutrophilic granulocytes; Lymph: lymphocytes; s-IAT: internet addiction total score; reference level for group was MDD.

DV: NR3C1 methylation

**Table S10.** Estimates, CIs and p-values for associations with NR3C1 methylation difference (t1-t0).

| *Predictors* | *β* | *CI* | *p* |
| --- | --- | --- | --- |
| (Intercept) | -0.0064 | -0.0258 – 0.0119 | 0.248 |
| NG | -0.0005 | -0.0081 – 0.0077 | 0.848 |
| Lymph | -0.0007 | -0.0099 – 0.0077 | 0.774 |
| BDI-II | -0.0061 | -0.0200 – 0.0091 | 0.076 |
| AD | 0.0025 | -0.0066 – 0.0122 | 0.361 |
| BMI | -0.0032 | -0.0116 – 0.0039 | 0.149 |
| Nicotine | 0.0046 | -0.0035 – 0.0123 | 0.049 |
| s-IAT | 0.0003 | -0.0083 – 0.0093 | 0.909 |
| Group | 0.0117 | -0.0167 – 0.0447 | 0.143 |
| s-IAT × Group | 0.0026 | -0.0124 – 0.0195 | 0.516 |

Note. BMI: Body Mass Index; BDI-II: baseline depression severity; AD: baseline dose equivalents of antidepressants; NG: neutrophilic granulocytes; Lymph: lymphocytes; s-IAT: internet addiction total score; reference level for group was MDD.

Social media use

DV: SLC6A4 methylation

**Table S11.** Estimates, CIs and p-values for associations with SLC6A4 methylation difference (t1-t0).

| *Predictors* | *β* | *CI* | *p* |
| --- | --- | --- | --- |
| (Intercept) | 0.0111 | -0.0160 – 0.0404 | 0.098 |
| NG | -0.0010 | -0.0111 – 0.0110 | 0.724 |
| Lymph | -0.0012 | -0.0126 – 0.0104 | 0.689 |
| BDI-II | -0.0007 | -0.0224 – 0.0172 | 0.887 |
| AD | -0.0013 | -0.0136 – 0.0121 | 0.716 |
| BMI | 0.0014 | -0.0116 – 0.0150 | 0.682 |
| Nicotine | 0.0020 | -0.0093 – 0.0130 | 0.558 |
| BSMAS | -0.0010 | -0.0140 – 0.0144 | 0.819 |
| Group | -0.0044 | -0.0463 – 0.0367 | 0.672 |
| BSMAS × Group | 0.0013 | -0.0210 – 0.0219 | 0.824 |

Note. BMI: Body Mass Index; BDI-II: baseline depression severity; AD: baseline dose equivalents of antidepressants; NG: neutrophilic granulocytes; Lymph: lymphocytes; BSMAS: Bergen Social Media Addiction Scale sum score; reference level for group was MDD.

DV: NR3C1 methylation

**Table S12.** Estimates, CIs and p-values for associations with NR3C1 methylation difference (t1-t0).

| *Predictors* | *β* | *CI* | *p* |
| --- | --- | --- | --- |
| (Intercept) | -0.0058 | -0.0265 – 0.0122 | 0.293 |
| NG | -0.0001 | -0.0075 – 0.0070 | 0.944 |
| Lymph | -0.0007 | -0.0087 – 0.0079 | 0.739 |
| BDI-II | -0.0066 | -0.0182 – 0.0053 | 0.047 |
| AD | 0.0028 | -0.0079 – 0.0130 | 0.278 |
| BMI | -0.0031 | -0.0113 – 0.0044 | 0.168 |
| Nicotine | 0.0053 | -0.0033 – 0.0145 | 0.022 |
| BSMAS | 0.0002 | -0.0110 – 0.0104 | 0.931 |
| Group | 0.0099 | -0.0196 – 0.0361 | 0.214 |
| BSMAS × Group | 0.0035 | -0.0111 – 0.0215 | 0.424 |

Note. BMI: Body Mass Index; BDI-II: baseline depression severity; AD: baseline dose equivalents of antidepressants; NG: neutrophilic granulocytes; Lymph: lymphocytes; BSMAS: Bergen Social Media Addiction Scale sum score; reference level for group was MDD.
